# Cannabidiol (CBD) Acts as an Antioxidant on *Gardnerella vaginalis*, Resulting in Reduced Metabolic Activity, Loss of Survivability, and Elimination of Biofilms

**DOI:** 10.3390/antibiotics14020136

**Published:** 2025-02-01

**Authors:** Ronit Vogt Sionov, Maya Korem, Itzhack Polacheck, Doron Steinberg

**Affiliations:** 1 Institute of Biomedical and Oral Research (IBOR), Faculty of Dental Medicine, The Hebrew University of Jerusalem, Jerusalem 9112102, Israel; dorons@ekmd.huji.ac.il; 2 Department of Clinical Microbiology and Infectious Diseases, Hadassah-Hebrew University Medical Center, Jerusalem 9112001, Israel; mayak@hadassah.org.il (M.K.); itzhackp@ekmd.huji.ac.il (I.P.)

**Keywords:** antibacterial, antibiofilm, antioxidants, bacterial vaginosis (BV), cannabidiol (CBD), *Gardnerella vaginalis*

## Abstract

**Background**: *Gardnerella vaginalis* is a natural inhabitant of the vagina, but when an imbalance occurs in the vaginal microbiota, this bacterium can cause vaginosis, a condition that must be treated when symptomatic and prior to a gynecological intervention. Cannabidiol (CBD) is an anti-inflammatory compound that also has antibacterial activities against several Gram-positive and certain Gram-negative bacteria. **Objectives**: Since *G. vaginalis* is an opportunistic pathogenic Gram-variable bacterium, we investigated its response to CBD. **Methods**: The antibacterial activity of CBD was studied by broth dilution assay, changes in intracellular ATP levels, and the ability of bacteria to recover on chocolate agar plates. The antibiofilm activity was investigated by MTT metabolic assay, crystal violet staining, and HR-SEM. Flow cytometric analyses were performed to measure changes in membrane potential, membrane perforation, and metabolic activity. Reactive oxygen species (ROS) production was analyzed using the nitro blue tetrazolium (NBT) reagent. Gene expression was determined by semi-quantitative real-time PCR, while protein composition was determined by LC-MS/MS analysis. **Results**: We observed that *G. vaginalis* clinical isolates exhibited high susceptibility to CBD with a minimum inhibitory concentration (MIC) of 2.5 µg/mL CBD. CBD induced rapid membrane hyperpolarization and caused cytoplasmic leakage of ATP without increasing propidium iodide uptake. This was accompanied by reduced metabolic activity and loss of survivability. Proteomic analysis revealed decreased expression of some ribosomal-associated proteins. CBD exhibited antioxidant activity by reducing intracellular ROS levels in a dose-dependent manner. The antibacterial effect was neutralized by the free radical scavenger α-tocopherol, suggesting the involvement of radicals in executing the antibacterial effect. Importantly, CBD not only prevented the biofilm formation of *G. vaginalis* but also reduced the metabolic activity and biofilm biomass of preformed, mature biofilms. Real-time PCR analysis of *G. vaginalis* treated with CBD for 6 h showed an increase in the expression of biofilm-associated genes, suggesting that the antibiofilm activity of CBD is mainly due to its antibacterial effect. CBD did not alter the ability of *G. vaginalis* to adhere to HeLa cervical carcinoma cells and CBD-treated bacteria were still phagocytosed by RAW264.7 macrophages. **Conclusions**: Our study shows that CBD exhibits antibacterial and antibiofilm activities against *G. vaginalis* clinical isolates and is thus a potential drug for the treatment of vaginosis caused by this bacterium.

## 1. Introduction

*Gardnerella vaginalis* (formerly known as *Haemophilus vaginalis*) is a Gram-variable, facultative anaerobic bacterium that is part of the normal microbiota of the vagina [1]. However, when an imbalance of the vaginal microbiota occurs, the excessive growth of *Gardnerella* and other anaerobic pathogenic bacteria such as *Prevotella*, *Porphyromonas*, *Bacteroides*, *Peptostreptococcus*, *Atopobium*, *Megasphaera*, and *Mobiluncus* can cause bacterial vaginosis (BV), which must be treated when symptomatic and prior to a gynecological intervention [2,3,4,5,6,7]. Among the 30 BV-associated species tested, *G. vaginalis* was found to be the most common and virulent species [8,9]. Vaginal dysbiosis has been associated with preterm birth, miscarriage, postpartum sepsis, neonatal infections, pelvic inflammatory disease, and an increased risk of human immunodeficiency virus (HIV) and human papillomavirus (HPV) infections [10,11,12,13,14]. The vaginal microbiota is affected by the hormone estrogen, the menstrual cycle, lifestyle, diet, and medications and it undergoes changes over the course of a woman’s life [3,5,15,16].

A healthy vaginal flora is characterized by the presence of various *Lactobacillus* species including *L. crispatus*, *L. gasseri*, *L. jensenii*, and *L. iners* [5,6,17]. These bacteria are important for maintaining a low pH (3.5–4.5) in the vagina by producing lactic acid through the fermentation of glucose and glycogen, among others [18,19,20]. In addition, these bacteria produce metabolites such as bacteriocins (e.g., lactocidin, acidolin, and lactacin B) and hydrogen peroxide, which inhibit the colonization of pathogenic bacteria such as *G. vaginalis*, *Chlamydia trachomatis*, and *Neisseria gonorrhoeae* and of the protozoan parasite *Trichomonas vaginalis* [5,18,19,21,22]. Lactic acid also exerts an antibacterial effect by acidifying the cytosol and increasing membrane permeability [22,23]. When the abundance of protective *Lactobacillus* species is reduced, the pH of the vagina increases, making this niche proficient for bacterial vaginosis (BV) caused by *G. vaginalis* and other anaerobic bacteria [1,5,22,24].

*G. vaginalis* has the ability to bind to the vaginal epithelium and displace *Lactobacilli* [25,26]. It is considered to be an early colonizer of the vagina preceding bacterial vaginosis which often has a multispecies nature [8,9,25,27]. Sialidase A production by *G. vaginalis* has been associated with biofilm formation and adhesion to epithelial cells and promotes the destruction of the protective mucus layer of the vaginal epithelium [28]. Within the biofilm, the bacteria are protected from lactic acid and hydrogen peroxide [29]. Moreover, *G. vaginalis* releases extracellular vesicles that carry bacterial proteins, which can be taken up by epithelial cells and stimulate the immune system [30].

Current treatment of vaginosis caused by *G. vaginalis* includes the antibiotics metronidazole and clindamycin [31,32]. However, metronidazole can also inhibit the growth of *Lactobacilli* spp. and thus hinder the restoration of a healthy vaginal environment [32]. Moreover, high recurrence rates (>50%) are observed after 6 months, which is attributed to factors such as antibiotic resistance, biofilm formation by *G. vaginalis* with lower sensitivity to antibiotics, and reinfection by the partner [32,33,34]. Lactic acid has been explored as a potential therapeutic approach to restore the vaginal microbiota and disrupt pathogenic biofilms [35]. Nevertheless, the current clinical efficacy of lactic acid-based treatments is still limited.

Cannabidiol (CBD), a non-psychoactive compound derived from the plant *Cannabis sativa* L., exhibits a range of medicinal properties [36]. Its anti-inflammatory effects can contribute to the attenuation of autoimmune diseases (e.g., multiple sclerosis, systemic lupus erythematosus, and rheumatoid arthritis) [37]. CBD also possesses antinociceptive and neuroprotective properties and is approved by FDA for the treatment of muscle spasms in multiple sclerosis, neuropathic pain, and seizures in refractory epileptic children [37]. As an antioxidant, CBD may play a role in preventing the progression of neurodegenerative diseases such as Alzheimer’s and Parkinson’s [38,39]. Additionally, CBD has been shown to have an antibacterial effect against various Gram-positive and certain Gram-negative bacteria, with minimum inhibitory concentrations (MICs) generally in the range of 1 to 5 μg/mL [40,41,42,43,44,45,46,47,48,49,50]. Importantly, the development of bacterial resistance to CBD appears to be low [40]. Interestingly, *Lactobacillus* species appear to be relatively resistant to the antibacterial effect of CBD. This is based on the observation of an increased relative abundance of *Lactobacillus* spp. in the feces of mice treated with CBD [51] and on the finding that *Cannabis sativa* L. extract exhibited antibacterial and antibiofilm activity against *Staphylococcus aureus* without inhibiting the growth of *Lactobacillus* spp. [52].

To date, there is no documentation in the literature on the effect of CBD on the Gram-variable *G. vaginalis*. We therefore studied the viability of *G. vaginalis* clinical isolates following CBD treatment and observed that these bacteria are highly susceptible to CBD, as evidenced by a loss of survivability with no regrowth of the bacteria when reseeded on chocolate agar plates. Exposure of *G. vaginalis* to CBD led to membrane hyperpolarization, cytoplasmic leakage of ATP, and a concomitant decrease in metabolic activity. Notably, the free radical scavenger α-tocopherol neutralized CBD’s antibacterial effect, suggesting that free radical generation plays a crucial role in its antibacterial mechanism. Importantly, CBD effectively reduced the viability of preformed, mature *G. vaginalis* biofilms, highlighting its potential as a novel therapeutic agent for the treatment of vaginosis caused by this bacterium.

## 2. Results

### 2.1. Cannabidiol (CBD) Is Bactericidal to Gardnerella vaginalis Clinical Isolates

**Figure 1:**
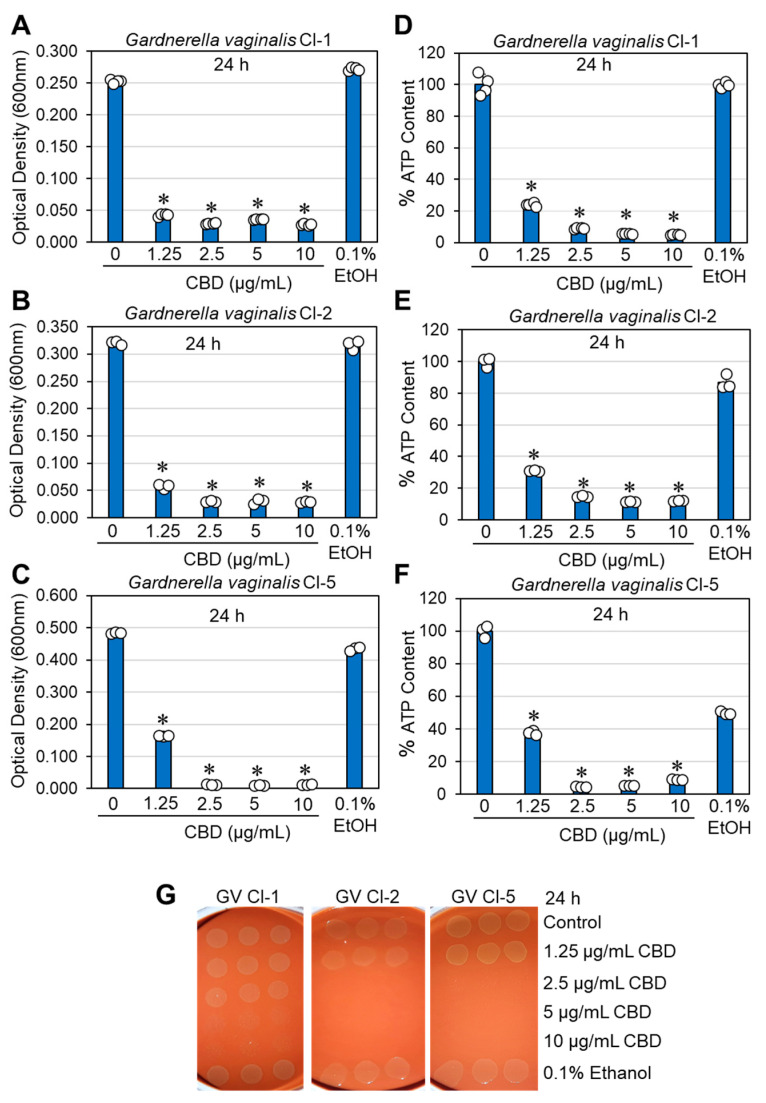
Antibacterial effect of CBD on *G. vaginalis* clinical isolates. *G. vaginalis* at an initial OD_600nm_ of 0.25 was incubated anaerobically in the absence or presence of cannabidiol (CBD) or 0.1% ethanol (EtOH) for 24 h in 10 mL BHI at 37 °C. (**A**–**C**). 10 mL of the bacterial culture was centrifuged and resuspended in 1 mL fresh medium to measure the turbidity at 600 nm in a flat-bottomed 24-well plate using a microplate reader. (**D**–**F**). The ATP content of the bacteria from 1 mL of cultures was determined by using the BacTiter-Glo microbial cell viability reagent. (**G**). Bacterial drop viability assay. At the end of incubation, 10 mL of the bacterial cultures was centrifuged and the bacterial pellet was resuspended in 1 mL of fresh BHI. Then, 10 μL of the concentrated bacterial samples was spotted onto chocolate blood agar plates, which were then incubated anaerobically for 48 h. N = 3–4; * *p* < 0.05 of treated samples compared to control and 0.1% ethanol. The individual data are presented in open circles.

**Figure 2:**
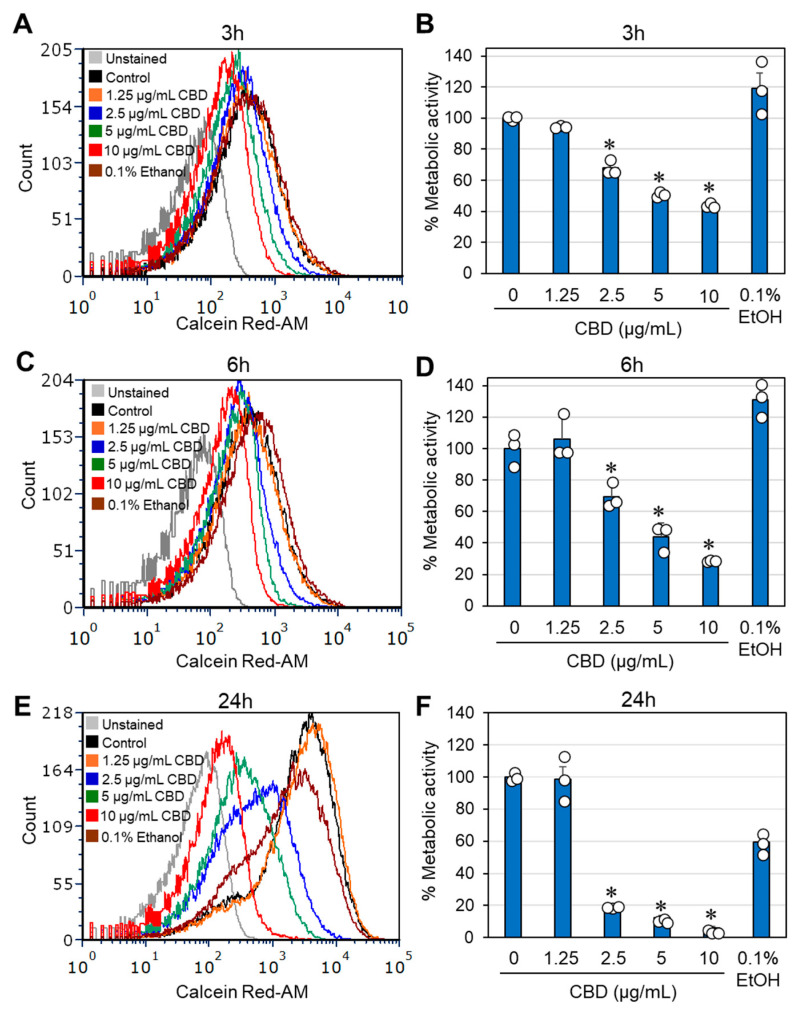
CBD reduces the metabolic activity of *G. vaginalis* CI-1. (**A**–**F**). Flow cytometric analysis of *G. vaginalis* that were incubated in the absence or presence of CBD for 3 h (**A**,**B**), 6 h (**C**,**D**) and 24 h (**E**,**F**) and labeled with 5 µM Calcein Red-AM during the last hour of incubation. Calcein Red-AM is cleaved into a red fluorescent compound in metabolically active bacteria. A total of 50,000 events were collected for each sample performed in triplicates. (**A**,**C**,**E**) are the respective histograms at the three time points. The shift of the histograms to the left is an indication for reduced metabolic activity. (**B**,**D**,**F**) present the relative fluorescence intensities (RFI) of the samples in (**A**,**C**,**E**), respectively. N = 3; * *p* < 0.05 of treated samples compared to control and 0.1% ethanol. The individual data are presented in open circles.

The effects of CBD on bacterial growth and viability were studied on the three clinical isolates of *G. vaginalis*. In terms of optical density, the minimum inhibitory concentration (MIC) resulting in no visible growth after a 24 h of incubation was determined to be between 1.25 and 2.5 μg/mL CBD (Figure 1A–C). Similar results were obtained for bacteria cultured in BHI, BHI/Wilkins–Chalgren at a 1:1 ratio, or Wilkins–Chalgren broth, whether the incubation volume was 300 μL or 10 mL. The relative ATP content of the CBD-treated bacteria was reduced by 85–95% after a 24 h of incubation when compared to control bacteria (Figure 1D–F), which is in accordance with the reduced bacterial turbidity (Figure 1A–C). To determine the viability of bacteria after a 24 h incubation with CBD, bacteria that were cultured in 10 mL of BHI with an initial OD_600nm_ of 0.25 were centrifuged, resuspended in 1 mL of fresh BHI, and 10 μL was spotted on chocolate blood agar plates for a 48 h incubation. The bacterial drop assay showed no bacterial growth at 5 and 10 μg/mL CBD for all three clinical isolates and no growth for clinical isolates 2 and 5, even at 2.5 μg/mL CBD (Figure 1G). This is in contrast to controls and bacteria exposed to 1.25 μg/mL CBD, where bacterial growth was observed on chocolate blood agar plates (Figure 1G). These data indicate that CBD is bactericidal to *G. vaginalis* at 5–10 μg/mL, while bacteriostatic at 1.25–2.5 μg/mL. A longer incubation time (5 days) with CBD showed similar growth profile with no growth recovery at MIC and higher concentrations (Supplementary Figures S1 and S2). To further prove the antibacterial activity of CBD on *G. vaginalis*, we analyzed the metabolic activity of control and CBD-treated bacteria after 3, 6, and 24 h of incubation using the Calcein Red-AM probe. Flow cytometry showed a 32–56% reduction in metabolic activity after 3 h, a 30–72% reduction after 6 h, and an 82–95% reduction after a 24 h of incubation in a dose-dependent manner after exposure to 2.5–10 µg/mL CBD (Figure 2A–F). The reduced metabolic activity corresponds with the reduced viability of the bacteria (Figure 1).

### 2.2. Cannabidiol (CBD) Causes ATP Leakage from the Bacteria

**Figure 3:**
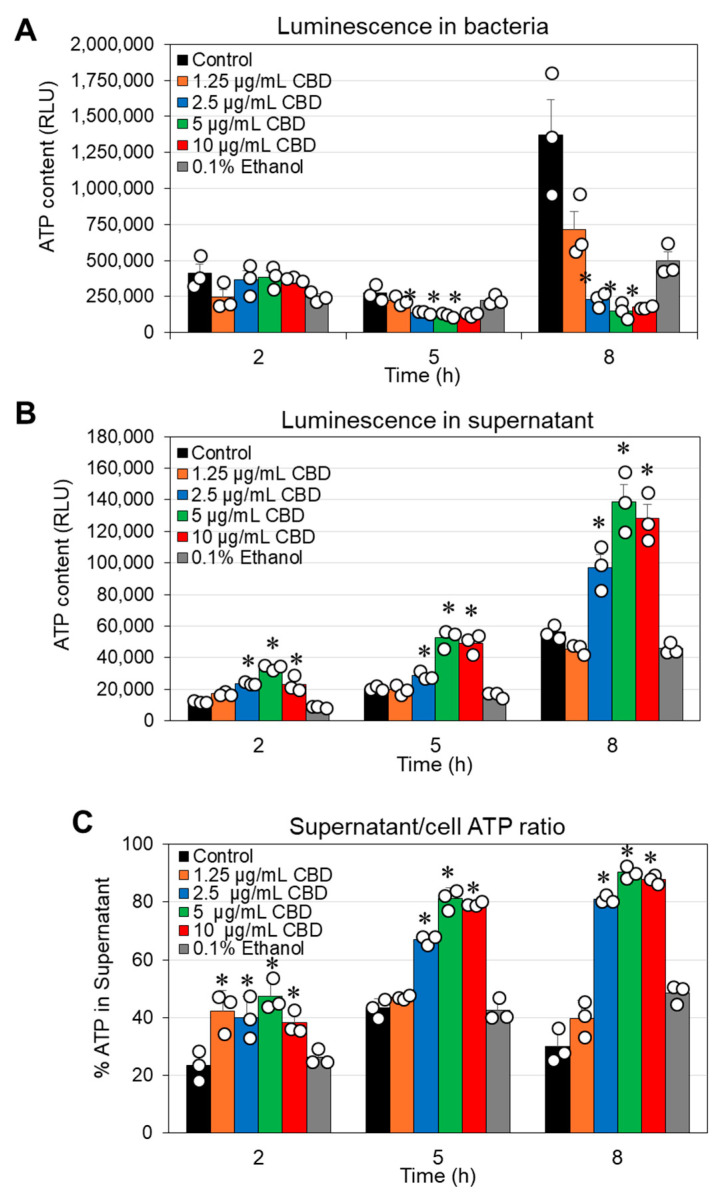
CBD causes ATP leakage from *G. vaginalis* CI-1 (**A**,**B**). The ATP content in *G. vaginalis* cells (**A**) and in the respective supernatants (**B**) was measured by BacTiter-Glo assay at various time points (2, 5 and 8 h) after exposing the bacteria to the indicated concentrations of CBD, 0.1% ethanol or the medium alone (control). Bacteria from 1 mL of culture were separated from the supernatant by centrifugation and resuspended in 100 µL of fresh medium to which 100 µL of the BacTiter-Glo solution was added (**A**). Then, 100 µL of the supernatant was mixed with 100 µL of BacTiter-Glo solution (**B**). (**C**). The ratio of ATP in the supernatant versus the cells in the samples presented in (**A**,**B**). RLU = relative luminescence units. N = 3. * *p* < 0.05 of treated samples compared to control and 0.1% ethanol. The individual data are presented in open circles.

Considering the reduced metabolic activity described above, it was inquiring to investigate the effect of CBD on the cytoplasmic leakage of ATP. To this end, we performed a kinetic study in which the bacteria were exposed to various concentrations of CBD for different durations. At the end of each incubation, the bacteria were separated from the supernatant and the luminescence emitted after adding the BacTiter-Glo reagent to the cells or the supernatants was recorded. The ATP content in the bacterial samples treated with 2.5–10 µg/mL CBD was significantly lower after a 5 h and 8 h incubation compared to control samples (Figure 3A), which may be due to reduced number of metabolically active bacteria. This is consistent with the flow cytometric analysis of CBD-treated bacteria using Calcein Red-AM as the metabolic probe (Figure 2). Notably, there was a concomitant increase in ATP in the supernatant (Figure 3B,C). These data suggest that CBD treatment of *G. vaginalis* leads to cytoplasmic leakage of ATP. However, there was no cytoplasmic leakage of the nucleic acid stain SYTO 9 (Supplementary Figure S3) and no appearance of a propidium iodide (PI)^high^ cell population (Supplementary Figure S3). These observations exclude the possibility that CBD causes membrane perforation. The DNA content per bacterium was only slightly reduced (20–30% reduction) after a 24 h incubation with 5 and 10 µg/mL CBD, as shown by DAPI staining of fixed bacteria (Supplementary Figure S4).

### 2.3. Morphological Studies of Gardnerella vaginalis Exposed to CBD

**Figure 4:**
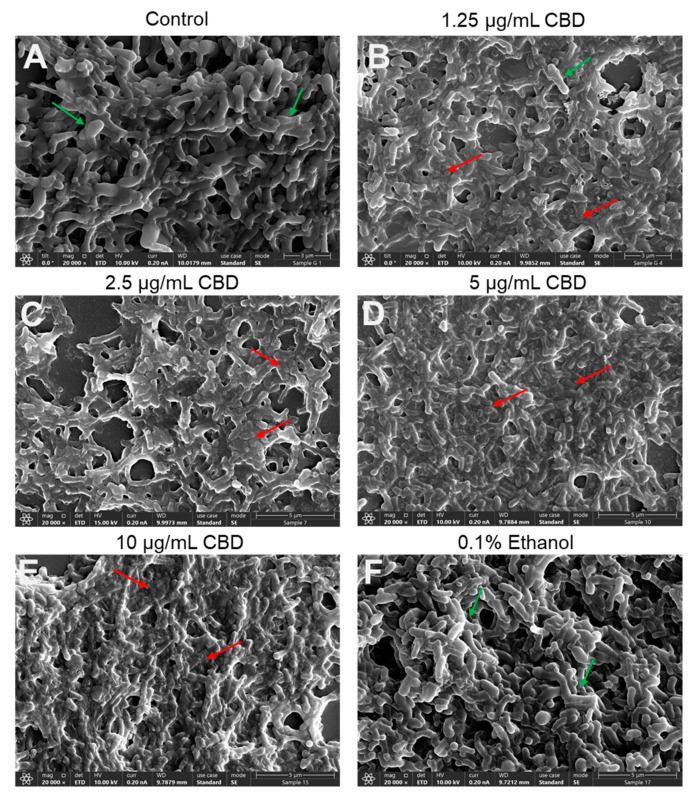
HR-SEM images showing the morphology of *G. vaginalis* CI-1 after a 24 h incubation with various concentrations of CBD. (**A**). Control; (**B**). 1.25 μg/mL CBD; (**C**). 2.5 μg/mL CBD; (**D**). 5 μg/mL CBD; (**E**). 10 μg/mL CBD; and (**F**). 0.1% ethanol. The green arrows point to live bacteria with lucid 3D structures, while the red arrows point to dead bacteria with flat and fragmented structures and the loss of cell boundaries. ×20,000 magnification.

To further verify the antibacterial action of CBD on *G. vaginalis*, we inspected its morphology by HR-SEM after a 24 h of incubation. The control and 0.1% ethanol-treated bacteria appeared as curved rods of various sizes, some of which were larger, whereas others were smaller (Figure 4A,F; Supplementary Figure S5A,F), which may represent different stages of cell division. The control and 0.1% ethanol bacteria appeared with clear 3D structures and smooth surfaces (Figure 4A,F; Supplementary Figure S5A,F). The CBD-treated bacteria, however, were flat and shriveled and had lost boundaries with diffuse and wrinkled membranes shared by neighboring bacteria (Figure 4B–E; Supplementary Figure S5B–E). Only occasionally were 3D bacterial structures with smooth surfaces observed (Figure 4B–E; Supplementary Figure S5B–E). The fact that we still observed bacteria after a 24 h exposure to CBD suggests that the bacteria did not undergo immediate autolysis despite the loss of viability. An interesting phenomenon was the frequent appearance of large structures after CBD treatment, which may be swollen bacteria or a fusion of several bacteria (Supplementary Figure S5B–E).

### 2.4. CBD Induces Immediate Membrane Hyperpolarization of Gardnerella vaginalis

**Figure 5:**
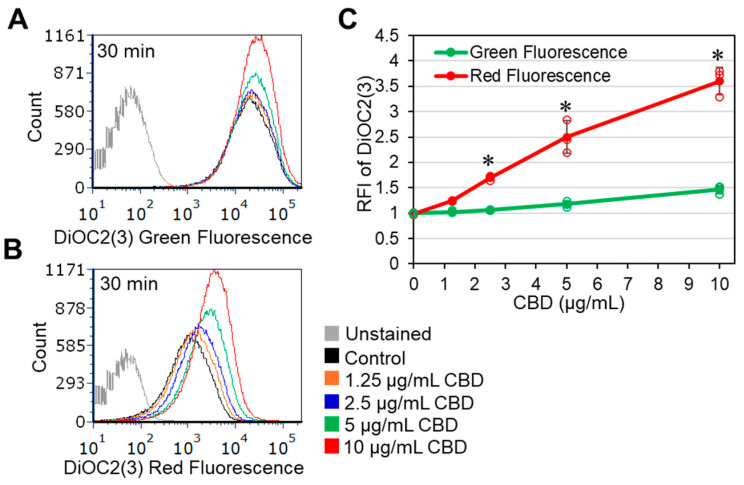
CBD causes immediate membrane hyperpolarization of *G. vaginalis*. *G. vaginalis* CI-1 was exposed to various concentrations of CBD in PBS for 10 min before adding the potentiometric dye DiOC2(3) for another 20 min. (**A**). Histograms of the green DiOC2(3) fluorescence intensities. (**B**). Histograms of the red DiOC2(3) fluorescence intensities. (**C**). The relative fluorescence intensities (RFI) of green and red emissions, showing a relative increase in red versus green fluorescence, indicative for membrane hyperpolarization. * *p* < 0.05 of the treated samples compared to the control samples. The individual data are presented in open circles.

In a search for the mode of action, the effect of CBD on the membrane potential was studied using the potentiometric dye DiOC2(3). We observed that CBD induced membrane hyperpolarization in a dose-dependent manner within 20 min, which was manifested by an increase in the red-to-green fluorescence ratio of DiOC2(3) on flow cytometry (Figure 5A–C). There was also a dose-dependent increase (1.4–1.8-fold) in Nile Red staining after 4 and 24 h of incubation (Supplementary Figure S6), suggesting an increase in the membrane content or an alteration in the membrane properties.

### 2.5. The Antibacterial Effect of CBD Is Neutralized by the Radical Scavenger α-Tocopherol

**Figure 6:**
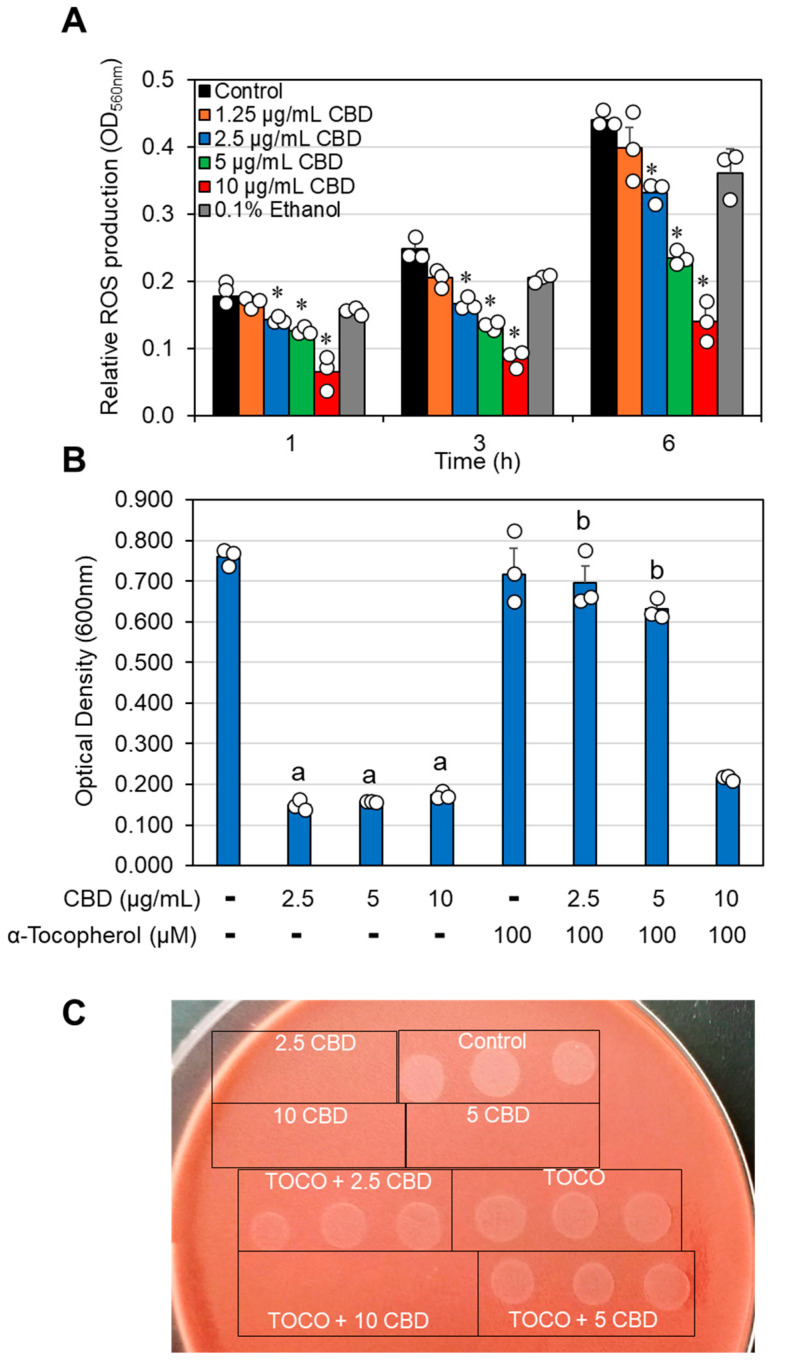
CBD acts as an antioxidant and its antibacterial activity is neutralized by the free radical scavenger α-tocopherol. (**A**). *G. vaginalis* CI-1 was loaded with the ROS sensor nitro blue tetrazolium (NBT) and then exposed to various concentrations of CBD or 0.1% ethanol for the indicated time periods. N = 3; * *p* < 0.05 of treated samples compared to control and 0.1% ethanol. (**B**). *G. vaginalis* CI-1 was exposed to the indicated concentrations of CBD in the absence or presence of the free radical scavenger α-tocopherol for 24 h and then the turbidity was measured at 600 nm. N = 3; “a” indicates *p* < 0.05 of CBD-treated samples compared to control samples. “b” indicates *p* < 0.05 of α-tocopherol-treated samples compared to the respective CBD-treated samples. (**C**). Bacterial drop viability assay of samples from (**B**). TOCO = α-tocopherol. The numbers in subfigure (**C**) represent the concentration in µg/mL. The individual data are presented in open circles.

CBD has been documented to affect reactive oxygen species (ROS) production in mammalian cells, resulting in both pro- and antioxidant effects [53,54,55]. It was therefore inquiring to examine how CBD affects ROS production in *G. vaginalis*. To this end, the bacteria were loaded with the ROS probe nitro blue tetrazolium (NBT) and exposed to various concentrations of CBD for 1, 3, and 6 h. The amount of NBT converted into formazan, which reflects ROS production, was dose-dependently reduced by CBD, reaching 70% inhibition at 10 µg/mL CBD at all tested time points (Figure 6A). These findings suggest that CBD acts as an antioxidant agent. Since the neutralization of ROS by antioxidants can generate radicals via the antioxidant itself [55,56], we wondered whether this could be the case for the antibacterial effect of CBD. We therefore applied the radical scavenger α-tocopherol and observed that it neutralized the antibacterial effect of CBD against *G. vaginalis* (Figure 6B). α-Tocopherol also rescued the loss of survivability caused by CBD (Figure 6C), suggesting that radical production by CBD is a major mode of its antibacterial action.

Since α-tocopherol has been shown to inhibit free radical-mediated lipid peroxidation [57], we tested whether CBD induces lipid peroxidation in *G. vaginalis*. To this end, we used two different techniques: The C11-Bodipy lipid peroxidation sensor, which undergoes a shift in its fluorescence emission from red to green upon oxidation [58], and a lipid peroxidation detection kit that is based on the production of malondialdehyde. Neither of the assays detected lipid peroxidation following CBD treatment of *G. vaginalis* (Supplementary Figure S7 and unpublished data), suggesting that another radical-mediated antibacterial mechanism is involved.

### 2.6. CBD Prevented Biofilm Formation and Destroyed Preformed Biofilms of Gardnerella vaginalis

**Figure 7:**
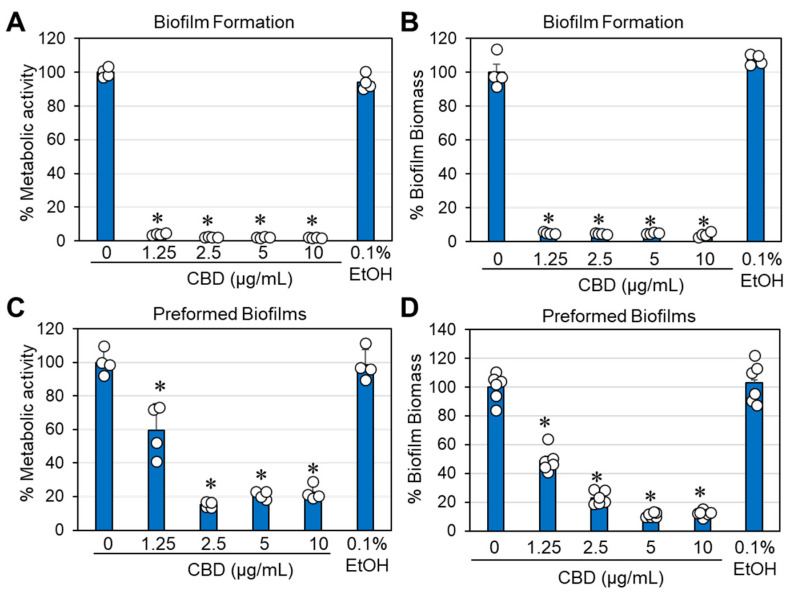
CBD prevents biofilm formation and destroys already formed biofilms of *G. vaginalis*. (**A**,**B**). Effect of CBD on biofilm formation by *G. vaginalis* CI-1. After a 3-day incubation of *G. vaginalis* CI-1 with an initial OD_600nm_ of 0.3 in 50% BHI/50% Wilkins Chalgren broth supplemented with 1% D-glucose, the biofilms formed in 24-well plates were either exposed to 1 mg/mL MTT for 3 h under anaerobic conditions (**A**, metabolic activity) or stained with crystal violet for 20 min (**B**, biofilm biomass). N = 4; * *p* < 0.05 of the treated samples compared to the control and 0.1% ethanol. (**C**,**D**). Effect of CBD on preformed biofilms. *G. vaginalis* CI-1 was allowed to form biofilms for 3 days while cultured in 1 mL 50% BHI/50% Wilkins–Chalgren broth containing 1% glucose in 24-well plates at an initial OD_600nm_ of 0.3. The biofilms were then treated with CBD at indicated concentrations or 0.1% ethanol as a control for 3 days. The metabolic activity of the resulting biofilms was determined by MTT metabolic assay (**C**) and the biofilm biomass determined by crystal violet staining (**D**). N = 4–6; * *p* < 0.05 of the treated samples compared to the control and 0.1% ethanol. The individual data are presented in open circles.

**Figure 8:**
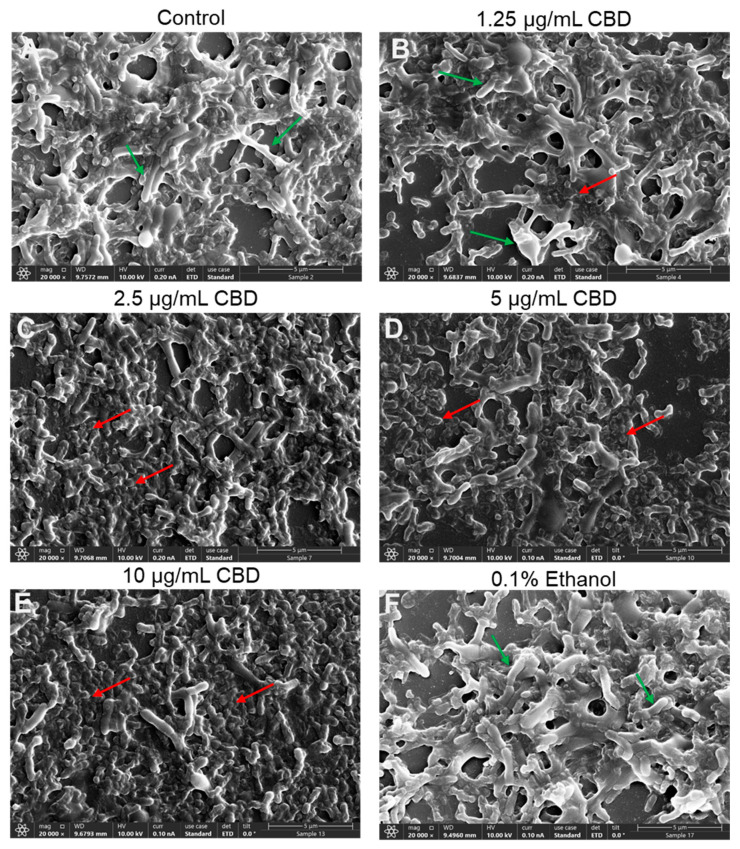
HR-SEM images of 3 day-preformed (mature) biofilms of *G. vaginalis* CI-1 that were exposed to CBD at various concentrations, 0.1% ethanol or medium alone (control) for 8 h. (**A**). Control; (**B**). 1.25 μg/mL CBD; (**C**). 2.5 μg/mL CBD; (**D**). 5 μg/mL CBD; (**E**). 10 μg/mL CBD; and (**F**). 0.1% ethanol. The green arrows point to live bacteria with lucid 3D structures, while the red arrows point to dead bacteria with flat and fragmented structures and the loss of cell boundaries. Magnification: ×20,000.

An important requirement for the successful treatment of *G. vaginalis* is the ability of the drug not only to prevent biofilm formation but also to act on preformed mature biofilms. We therefore investigated whether CBD could both prevent biofilm formation and reduce preformed mature biofilms of *G. vaginalis*. The biofilm formation assay endured for three days, which is the time required to form mature biofilms. To study the effect of CBD on mature biofilms, these were allowed to form for three days prior to CBD treatment. We observed that CBD prevented biofilm formation with a minimum biofilm inhibitory concentration (MBIC) of 1.25 μg/mL (Figure 7A,B) and reduced the metabolic activity and biofilm biomass of mature biofilms by 78–80% at 2.5 μg/mL CBD and by 88–90% at 5 and 10 μg/mL CBD after a 3-day incubation (Figure 7C,D). HR-SEM images of mature biofilms exposed to 2.5–10 μg/mL CBD for 8 h showed that the majority of bacteria had lost their vital 3D structure and appeared as flat, rounded up bacteria that had lost lucidity (Figure 8C–E). This is in contrast to the control bacteria, the 1.25 μg/mL CBD-treated, and 0.1% ethanol-treated bacteria, which appeared as vital 3D structures with smooth surfaces in the biofilm (Figure 8A,B,F).

### 2.7. CBD Increased the Expression of Biofilm-Related Genes in Gardnerella vaginalis

Gene expression studies of CBD-treated *G. vaginalis* showed rather an increase in the expression of glycosyltransferase (*gtf*) and the *flp* pilus-assembly TadE/G-like family protein (*pat*) after a 6 h incubation with CBD (Supplementary Figure S8A), suggesting that the antibiofilm activity of CBD is likely related to its bactericidal effect. Moreover, vaginolysin (*vly*) and some membrane transporters were upregulated by CBD (Supplementary Figure S8A). Notably, the *groEL* gene (chaperonin-60), which encodes for a heat shock protein (Chaperonin-60) involved in posttranslational protein folding and assembly, was significantly downregulated (Supplementary Figure S8A). The increased expression of biofilm-related genes may explain why CBD does not prevent the binding of *G. vaginalis* to HeLa cervical epithelial cells (Supplementary Figure S8B) and is still phagocytosed by RAW 264.7 macrophages (Supplementary Figure S9). Even a 4 h pretreatment of *G. vaginalis* with CBD did not alter the ability of the bacteria to bind to HeLa cells; nor did it affect their phagocytosis by RAW 264.7 macrophages.

### 2.8. Analysis of the Proteome Showed Reduced Expression of Some Ribosomal Components

Next, we wanted to know whether CBD has any effect on protein expression. To this end, *G. vaginalis* samples that were incubated in the absence or presence of 2.5 μg/mL and 5 μg/mL CBD or 0.5% ethanol for 6 h were subjected to total protein extraction and proteomic analysis. This analysis showed that twelve proteins were significantly downregulated and four proteins were significantly upregulated out of 386 proteins detected by LC-MS/MS analysis (Table 1). Outstanding was the 2–3-fold reduction in six ribosomal-related proteins: large ribosomal subunit protein bL12 (RplL), small ribosomal subunit protein uS7 (RpsG), 50S ribosomal protein L35 (RpmI), ribosome-recycling factor Frr, large ribosomal subunit protein bL34 (RpmH), and the RNA polymerase-binding protein RbpA. Among the significantly upregulated proteins, cysteine synthase was of special interest, as it might be a response to the distorted redox status of the bacteria caused by CBD.

Gene expression studies of selected ribosome-related genes of *G. vaginalis* that were exposed to CBD for 6 h surprisingly showed that the genes encoding for large ribosomal subunit protein bL12, 50S ribosomal protein L35, and RbpA were upregulated (Supplementary Figure S10), which might be due to a compensation mechanism for the reduced protein expression, or the reduced protein expression was due to a post-translational regulation. On the other hand, *rpsG* gene expression was downregulated, which goes along with the reduced protein expression (Table 1). However, there was no significant alteration in the *rpmH* gene level (Supplementary Figure S10). The discrepancies between proteomics and gene expression may be due to compensatory mechanisms or post-transcriptional regulation.

## 3. Discussion

This study aimed to investigate whether CBD has antibacterial effects against the pathogenic bacterium *G. vaginalis*, which is the major bacteria detected in BV and BV recurrence. CBD has already been shown to exert antibacterial activities against several Gram-positive bacteria (e.g., *Streptococcus* spp. and *Staphylococcus* spp.) and selected Gram-negative bacteria, including the vaginal pathogen *N. gonorrhoeae* [40,41,42,43,44,45,46,47,48,49,50]. In the presence of the LPS-binding drug polymyxin B, CBD could also act on the Gram-negative bacteria *Klebsiella pneumoniae*, *Escherichia coli*, *Pseudomonas aeruginosa*, and *Acinetobacter baumannii* [40,59,60]. A major advantage of CBD is its antibacterial action on both antibiotic-susceptible and antibiotic-resistant bacteria [45,46,50], suggesting that its mechanism of action is not affected by the common resistance mechanisms. Our study revealed that *G. vaginalis* is highly sensitive to CBD, leading to a loss of survivability already at concentrations as low as 2.5–5 µg/mL (Figure 1). Importantly, CBD not only prevented biofilm formation but also reduced preformed mature biofilms of *G. vaginalis* (Figure 7). Notably, the concentrations required for the antibacterial and antibiofilm activity of CBD on *G. vaginalis* are not toxic to Vero epithelial cells [43] and periodontal ligament fibroblasts [61]. These findings suggest that CBD may be a potential drug for the treatment of vaginosis caused by this bacterium. CBD is used in the clinic to treat certain brain disorders, such as refractory epileptic seizures in patients with Dravet, Lennox–Gastaut, and tuberous sclerosis complex (TSC) syndromes [62], and to relieve spastic symptoms in patients with multiple sclerosis [63].

The mechanisms of the antibacterial actions of CBD are only partially understood. Some studies have shown that CBD has an antimetabolic effect on *Streptococcus mutans* [44,45] with a general reduction in protein, DNA, RNA, and peptidoglycan synthesis in *S. aureus* [40]. Others have shown that CBD inhibits the release of membrane vesicles from *E. coli*, thereby altering their susceptibility to antibiotics [64]. However, this effect was not observed in Gram-positive bacteria [64]. Owing to its hydrophobicity, it has been anticipated that its antibacterial activity is due to nonspecific effects on the bacterial membrane [59], although so far there has not been any direct evidence of what these “nonspecific” effects are. In mammalian cells, CBD has been found to intercalate into cytoplasmic membranes, where it alters cholesterol homeostasis [65] and causes a rapid increase in intracellular calcium levels [65]. We and others have previously shown that CBD alters the bacterial membrane potential, resulting in membrane hyperpolarization in *S. mutans* [43], and membrane depolarization in *S. aureus* [40,50]. Likewise, we show here that CBD causes an immediate membrane hyperpolarization in *G. vaginalis* (Figure 5), suggesting that CBD has a specific effect on components involved in regulating the membrane potential. This finding is in line with the well-documented effect of CBD in modulating the activity of various ion channels in mammalian cells (e.g., [66,67,68,69]).

An interesting phenomenon is that *G. vaginalis* does not undergo immediate lysis after CBD treatment, as shown by the still presence of bacteria in the HR-SEM images (Figure 4), albeit that they appear with aberrant structures (Figure 4) and have lost survivability (Figure 1 and Supplementary Figure S2). There was no increase in propidium iodide uptake (Supplementary Figure S3), suggesting that the membranes were not perforated by CBD. Propidium iodide is a positively charged fluorescent dye that can only penetrate bacteria with damaged membranes and emits red fluorescence when bound to nucleic acids. However, there was a time-dependent and concentration-dependent leakage of ATP into the surroundings, which was accompanied by a concomitant decrease in intracellular ATP levels (Figure 3) and decreased metabolic activity (Figure 2). The immediate membrane hyperpolarization caused by CBD (Figure 5) may account for some of the antimetabolic effects, as the membrane potential is an important energy source for the bacteria through generating the proton motive force required for ATP production [70]. An antimetabolic effect of CBD has also been observed in *S. mutans* [44] and in the fungus *Candida albicans* [71]. *G. vaginalis* is a slow-growing bacterium with low metabolic activity, which may explain its higher sensitivity to CBD than the Gram-positive *S. mutans* and *S. aureus*, where bacterial subpopulations manage to resume growth after removing CBD from the medium, even at a concentration four times higher than the MIC value (unpublished data). The alterations in membrane polarization together with cytoplasmic leakage of ATP and reduced metabolic activity in *G. vaginalis* may account for the loss of survivability following CBD treatment. Extracellular ATP released from dying cells has been shown to act as a stress signal molecule that can recruit immune cells to eliminate damaged cells [72].

Another interesting finding was that the free radical scavenger α-tocopherol (vitamin E) prevented the antibacterial effect of CBD on *G. vaginalis* (Figure 6B,C). α-Tocopherol has previously been shown to counteract the antibacterial activity of the polyunsaturated fatty acid arachidonic acid against *S. mutans* [73] and *S. aureus* [57], among others, by inhibiting lipid peroxidation caused by radicals formed when arachidonic acid reacts with reactive oxygen species (ROS) [57]. We observed that CBD had antioxidant effects by reducing the ROS levels in *G. vaginalis* in a dose-dependent manner (Figure 6A), suggesting that CBD may interact with ROS. An antioxidant activity has been attributed to CBD in mammalian cells [53,54,55], which is partly due to a direct reaction of CBD with ROS [74,75] and through the regulation of antioxidant mechanisms [53,76]. In contrast to arachidonic acid, which causes lipid peroxidation when reacting with ROS [57], we could not detect any induction of lipid peroxidation by CBD in *G. vaginalis* (Supplementary Figure S7). CBD has even been shown to reduce lipid peroxidation in the brains of mice exposed to hypoxic conditions [77]. Thus, we can exclude lipid peroxidation as a mechanism for the antibacterial effect of CBD. When reacting with ROS, CBD can be oxidized to form cannabinoquinoids such as cannabidiolquinone [75], which are unstable compounds with the potential to generate radicals. The antibacterial action of the oxidized form of CBD has yet to be determined. Several antioxidants have been shown to have antibacterial effects [78], suggesting that there is a link between the antioxidant and antibacterial activities of these compounds.

Protein analysis of *G. vaginalis* treated with CBD for 6 h showed an increased expression of cysteine synthase (Table 1), which could be a compensatory mechanism to cope with the CBD-induced stress. This enzyme is involved in the production of the amino acid cysteine, which is a precursor of the glutathione biosynthesis pathway [79]. Glutathione in turn regulates the redox status of the cell [79]. Cysteine is also involved in the upregulation of ribosomal genes [80]. In this context, it is worth noting that several ribosome-related proteins were downregulated by CBD (Table 1), so the upregulation of cysteine synthase could help to counteract this stress. It is not known how CBD reduces the expression of ribosome-related proteins, and of the downregulated ribosomal proteins, only *rpsG* was significantly downregulated at the gene expression level (Supplementary Figure S10). The gene expression of rpmH remained unaffected by CBD, while the gene expression of *rplI* and *rbpA* was even upregulated (Supplementary Figure S10). This could again be due to a compensatory mechanism, or the expression of ribosomal proteins may be regulated by a posttranslational mechanism. Our findings are supported by a recent study by Machado et al. [81], who reported reduced ribosome biogenesis transcripts in the hippocampal subregions of mice treated with CBD. Overall, the reduced level of ribosomal proteins may account for some of the antibacterial activity of CBD against *G. vaginalis*.

Another property of CBD is its ability to prevent biofilm formation by *G. vaginalis* (Figure 7A,B). The minimum biofilm inhibitory concentration (BMIC) was found to be 1.25 µg/mL (Figure 7A,B), which is below the MIC of 2.5 µg/mL (Figure 1), suggesting that CBD has specific antibiofilm activity. However, gene expression studies did not show a downregulation but rather an upregulation of biofilm-related genes, suggesting that other mechanisms are involved in the antibiofilm activity. At the MIC and higher concentrations, the antibiofilm effect is mainly due to the antibacterial activity. Importantly, CBD was also effective against preformed mature biofilms of *G. vaginalis* (Figure 7C,D), which is an important consideration in clinical situations where these bacteria have already formed biofilms prior to diagnosis. The concentration required to eliminate the mature biofilms was close to the MIC. Already after an 8 h incubation with CBD, morphological changes could be observed by HR-SEM imaging (Figure 8). These findings suggest that CBD can penetrate through the *G. vaginalis* multilayered biofilm and kill the bacteria. Despite the reduced survivability of *G. vaginalis* after CBD treatment, the treated bacteria were still able to bind HeLa cervical epithelial cells (Supplementary Figure S8B) and be phagocytosed by macrophages (Supplementary Figure S9), which is an important process for eliminating bacteria.

**Figure 9:**
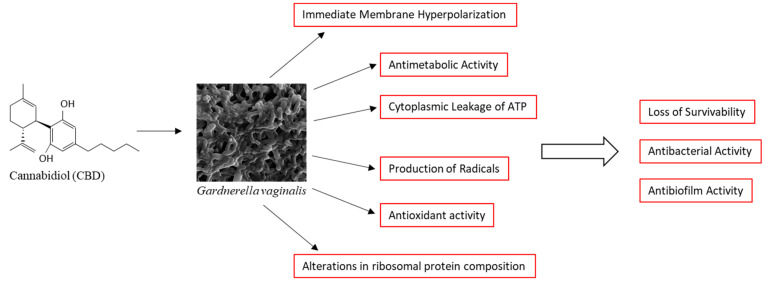
Proposed mode of action of CBD on *G. vaginalis*.

In conclusion, we present here data showing that CBD has antibacterial and antibiofilm activities against the Gram-variable *G. vaginalis*, which is an opportunistic pathogen associated with bacterial vaginosis. We provide mechanistic insights into the mode of action of CBD. CBD causes immediate membrane hyperpolarization, cytoplasmic leakage of ATP, production of cytotoxic radicals, alterations in ribosomal protein composition, and metabolic inhibition. These effects ultimately lead to the loss of survivability of the bacteria, which is a major underlying cause for the antibacterial and antibiofilm effects of CBD (Figure 9). The high susceptibility of *G. vaginalis* to CBD makes CBD a potential drug for the treatment of vaginosis caused by this bacterium. Since the MIC of CBD depends on the bacterial density (it increased from 1.25 to 2.5 μg/mL when the OD increased from 0.1 to 0.3, Supplementary Figure S1), we recommend CBD for the treatment of residual disease and for the prevention of relapse. The MIC value is still much lower than that required for a cytotoxic effect on the epithelium [43], which provides a sufficient therapeutic window. Furthermore, the administration of the drug is localized in the vagina, and any absorption of the drug into the blood system results in rapid degradation of CBD during the first passage in the liver [82], thereby limiting potential adverse effects. The limitation of this study is that it is an in vitro study. Further studies should focus on the in vivo efficacy of CBD in treating bacterial vaginosis and detecting any possible toxicity in animal models before conducting human clinical trials in the future.

## 4. Materials and Methods

### 4.1. Materials

CBD (purity > 99%, see Certificate of Analysis in Supplementary Data) was purchased from NC Labs (Prague, Czech Republic) and dissolved at 10 mg/mL in ethanol. α-Tocopherol was purchased from Sigma-Aldrich (St. Louis, MO, USA) and dissolved at 20 mM in ethanol. Equal ethanol concentrations (0.01–0.1%) were used in the experiments as controls. HPLC-grade ethanol was purchased from Baker (Gliwice, Poland).

### 4.2. Cultivation of Gardnerella vaginalis

Three *Gardnerella vaginalis* clinical isolates from vaginal samples were obtained from a collection stored at the Clinical Microbiology Laboratory of the Hadassah Medical Center, Jerusalem, Israel. Bacterial identification was performed via matrix-assisted laser desorption/ionization time-of-flight mass spectrometry (MALDI-TOF MS) (bioMérieux, Marcy l’Etoile, France) [83] and by quantitative real-time PCR using the following primers for *Gardnerella vaginalis* 16S rRNA: F: 5′-TGA GTA ATG CGT GAC CAA CC-3′/R: 5′-AGC CTA GGT GGG CCA TTA CC-3′ [84] and *Gardnerella vaginalis* vaginolysin: F: 5′-GAA CAG CTG GGC TAG AGG TG-3′/R: 5′-AAT TCC ATC GCA TTC TCC AG-3′ [85] on purified bacterial DNA. Bacterial DNA was isolated using the NucleoSpin Microbial DNA isolation kit (Machery-Nagel GmbH & Co, Düren, Germany).

The bacteria were inoculated on a blood agar base supplemented with 5% defibrinated sheep blood (Novamed, Jerusalem, Israel) or chocolate blood agar plates (Hylabs, Rehovot, Israel) and incubated at 37 °C under anaerobic conditions using AnaeroGen sachets (Thermo Scientific, Oxoid, UK). The bacteria formed small transparent, pinpoint-like, silver-grey colonies on chocolate blood agar (Supplementary Figure S11). *G. vaginalis* is a slow-growing bacterium, and therefore different culture conditions were initially used to determine the optimal growth conditions for the bacteria. Individual colonies were incubated under anaerobic conditions in 14 mL of brain–heart infusion (BHI) broth (HiMedia, Laboratories Pvt. Ltd., Maharashtra, India), Wilkins–Chalgren anaerobic broth (Oxoid Ltd. Bashingstoke, Hampshire, UK), or a 50:50 mixture of BHI and Wilkins medium in 15 mL tubes in a sealed anaerobic jar using an AnaeroGen sachet (Oxoid Ltd., Bashingstoke, Hampshire, UK). After 2–3 days of incubation, the bacteria were centrifuged at 5000× *g* for 10 min and the medium was replaced with fresh medium for another 24 h of incubation. This procedure was repeated until a sufficient number of bacteria was obtained. All three culture conditions were used to investigate the antibacterial activity of CBD, while 50% BHI/50% Wilkins broth supplemented with 1% D-glucose was used for the biofilm assays and RNA isolation. The other experiments were performed in BHI medium. All experiments were performed under anaerobic conditions, except at the endpoint reading step.

### 4.3. Determination of the Antibacterial Effect of Cannabidiol (CBD)

The antibacterial effect of CBD (1–10 µg/mL) on planktonic growing bacteria was initially studied by using the standard microbroth dilution assay in 96 flat-bottomed tissue-grade wells (Corning Incorporation, Kennebunk, ME, USA) [43]. Owing to slow bacterial growth, the antibacterial effect was repeatedly analyzed with different initial bacterial densities (optical density (OD) at 600 nm of 0.1–0.3) in a volume of 300 μL and for various time points (24, 48, and 72 h). At the end of the incubation period, the turbidity was determined by measuring the OD at 600 nm in a Multiskan SkyHigh microplate reader (Thermo Scientific, Life Technologies Holdings Pte Ltd., Singapore) [73]. Only after 3 days of incubation in the 96-well plates, the turbidity in the control and ethanol-treated samples was sufficient for accurately determining the extent of the antibacterial effect. We therefore adapted a modified antibacterial assay to validate the antibacterial effect of CBD: The bacteria were inoculated at an initial OD_600nm_ of 0.3 in 10 mL BHI broth and after a 24 h incubation the bacteria were precipitated by centrifugation at 5000× *g* for 10 min, resuspended in 1 mL fresh BHI medium, and 300 μL was transferred to each well of a 96-well plate for determining the OD_600nm_ in a microplate reader. The minimum inhibitory concentration (MIC) was determined as the lowest concentration of the agent causing no visible bacterial growth, meaning an OD_600nm_ of treated samples close to the OD_600nm_ of the background (medium alone). Both assays were repeated three times. The % turbidity was determined by the following formula:

% Turbidity = (OD_treated samples_ − OD_background_)/(OD_control_ − OD_background_) × 100%.

### 4.4. Survivability Assay

For determining the survivability of the bacteria from the 96-well plate microbroth dilution assay (Section 4.3), 10 μL from each sample in triplicates was inoculated on chocolate blood agar plates for 48 h under anaerobic conditions at 37 °C to visualize live bacteria. For the 10 mL cultures, the cultures were first centrifuged and the bacterial pellets were resuspended in 1 mL of fresh medium prior to spotting 10 μL from each sample on chocolate blood agar plates.

### 4.5. Determination of the ATP Content in Bacteria

Another assay used to determine the viability of bacteria was the BacTiter-Glo microbial cell viability reagent (Promega, Madison, WI, USA) which measures ATP content [86]. The BacTiter-Glo reagent causes bacterial cell lysis to release their ATP content, which is detected by the conversion of D-luciferin to oxyluciferin by recombinant Ultra-Glo luciferase, resulting in the emission of luminescence whose intensity is proportional to the amount of ATP. To this end, 1 mL of the cultures grown in 10 mL were centrifuged at 5000× *g* for 10 min at 4 °C and the bacterial pellet was resuspended in 100 μL of fresh medium, to which 100 μL of the BacTiter-Glo reagent was added. Following a 10 min incubation at room temperature, the luminescence was measured in a μ-clear 96-well flat-bottom white microplate (Greiner Bio-One GmbH, Frickenhausen, Germany) using an M200 infinite plate reader (Tecan Group Ltd., Männedorf, Switzerland). The % ATP content was determined using the following formula, where RLU is the relative luminescence units:

% ATP content = (RLU_treated samples_ − RLU_background_)/(RLU_control_ − RLU_background_) × 100%.

To study the amount of ATP leakage into the medium, 100 μL of the bacteria-free supernatant was mixed with 100 μL of the BacTiter-Glo reagent. The percentage of ATP in the supernatant was calculated by the following formula:

(RLU_supernatant_ × 10)/(RLU_bacterial cells_ + (RLU_supernatant_ × 10)) × 100%

where RLU_supernatant_ is the relative luminescence units in 100 µL supernatant out of 1 mL and RLU_bacterial cells_ is the relative luminescence units of bacteria from 1 mL culture. Media without bacteria that were mixed with BacTiter-Glo reagent served as background reads.

### 4.6. Determining the Metabolic Activity of Bacteria by Calcein Red-AM

Control and treated bacteria were centrifuged and resuspended in 1 mL of fresh BHI containing 5 µM Calcein Red-AM (Biolegend, San Diego, CA, USA), which is converted to a red fluorescent compound in metabolically active cells [87]. After a 1 h incubation, the bacteria were washed in PBS and the intensity of red fluorescence was measured in a LSR Fortessa flow cytometer (BD Biosciences, San Jose, CA, USA) with excitation/emission wavelengths of 561 nm/635 nm. A total of 50,000 events were collected for each sample in triplicate using the FACSDiva 8.0.1 software and the data were analyzed using the FCS Express 7.12.0007 software. The flow cytometric analysis has the advantage that it measures the fluorescence intensity of each individual bacterium. This allows the determination of the metabolic activity per bacterium from the entire bacterial population when using Calcein Red-AM as the probe. The histograms represent an integration of 50,000 events collected for each sample. The shift of the histogram to the left indicates lower metabolic activity.

### 4.7. Determination of the Antibiofilm Effects of CBD on Biofilm Formation and Preformed Biofilms

To study the effect of CBD on biofilm formation, *G. vaginalis* at an initial OD_600nm_ of 0.3 was incubated in 1 mL of 50% BHI/50% Wilkins broth and 1% D-glucose in the absence or presence of CBD (1.25–10 µg/mL) in 24-well flat-bottomed tissue culture grade plates for 3 days at 37 °C under anaerobic conditions. At the end of incubation, the biofilms were washed twice with PBS and then either stained with 500 μL of a 0.25% crystal violet (CV) solution (diluted 1:4 from 1% Gram crystal violet solution, Merck KGaH, Darmstadt, Germany) for 20 min at room temperature [43] or the metabolic activity was determined by incubating the biofilms with 500 μL of 1 mg/mL MTT (3-(4,5-dimethyl-2-thiazolyl)-2,5-diphenyl-2H-tetrazolium bromide; Sigma, St. Louis, MO, USA) for 3 h at 37 °C under anaerobic conditions [43]. The CV-stained biofilms were washed twice with double-distilled water (DDW) to remove excess staining solution and the biofilm staining was dissolved in 1 mL of a 33% acetic acid solution prior to measuring the absorbance at 595 nm in a Multiskan SkyHigh microplate reader. The percentage biofilm mass after CBD treatment was determined by the following formula:

% Biofilm Biomass = (OD_treated samples_ − OD_background_)/(OD_control_ − OD_background_) × 100%.

For the metabolic assay, the MTT solution was removed after the incubation and the formazan precipitates in the biofilms were dissolved in 1 mL dimethylsulfoxide (DMSO) prior to reading the absorbance at 570 nm in a plate reader. The percentage metabolic activity of biofilms after CBD treatment was determined by the following formula:

% Metabolic Activity = (OD_treated samples_ − OD_background_)/(OD_control_ − OD_background_) × 100%.

To study the effect of CBD on preformed (mature) biofilms, *G. vaginalis* was allowed to form biofilms by incubating the bacteria at an initial OD_600nm_ of 0.3 in 1 mL of 50% BHI/50% Wilkins broth supplemented with 1% D-glucose in 24-well flat-bottomed tissue culture grade plates for 3 days at 37 °C under anaerobic conditions. On the second day of incubation, half of the medium was replaced with fresh 50% BHI/50% Wilkins broth supplemented with 1% D-glucose. On the third day, the biofilms were washed gently with 1 mL of PBS and then incubated for 24–72 h in 50% BHI/50% Wilkins broth containing 1% D-glucose with various concentrations of CBD. At the end of incubation, the biofilms were stained with CV or the metabolic activity was determined by the MTT assay as described above.

### 4.8. Morphological Studies Using High-Resolution Scanning Electron Microscopy (HR-SEM)

To study the morphology of planktonically growing cells after a 24 h of incubation with CBD, the bacteria were washed once in DDW and fixed in 4% glutaraldehyde (Electron Microscopy Sciences, Hatfield, PA, USA) in DDW for 2 h, resuspended in 100 μL DDW, and 10–20 μL of the bacterial slurry were spotted on 0.7 cm × 0.7 cm glass pieces made from slides and allowed to dry [43]. *G. vaginalis* was allowed to form biofilms on glass pieces for 3 days as described in Section 4.7 and then exposed to CBD for 8 h to study the early changes in bacterial morphology. At the end of incubation, the biofilms were washed with DDW, fixed with 4% glutaraldehyde in DDW for 2 h, washed in DDW, and allowed to dry. The samples were coated with iridium and visualized by an analytical high-resolution scanning electron microscope (HR-SEM) (Apreo 2 S LoVac, Thermo Scientific) at various magnifications.

### 4.9. Determination of the Membrane Potential Using DiOC2(3) Potentiometric Dye

*G. vaginalis* was resuspended to an OD_600nm_ of 0.3 in PBS and 1 mL was taken for each sample. The bacteria were centrifuged at 5000× *g* for 5 min in an Eppendorf centrifuge and the pellet was resuspended in 1 mL of PBS with or without CBD (1.25–10 μg/mL). After a 10 min incubation, at room temperature, DiOC2(3) (3,3′-diethyloxacarbocyanine, iodide, BacLight Membrane Potential Kit, Molecular Probes, Life Technologies, Eugene, OR, USA) was added to each sample at a final concentration of 30 µM. The samples were incubated for 20 min, filtrated through a cell strainer, and the relative fluorescence intensities (RFIs) were analyzed in a LSR Fortessa flow cytometer (BD Biosciences) using the excitation/emission wavelengths of 488 nm/530 nm for green fluorescence and 488 nm/620 nm for red fluorescence [43]. A relative increase in red fluorescence in comparison to green fluorescence is an indication of membrane hyperpolarization. A total of 50,000 events were collected for each sample using FACSDiva 8.0.1 software and the collected data were analyzed using FCS Express 7.12.0007 software. DiOC2(3) is a potentiometric green fluorescent dye that penetrates the bacterial membrane and forms red fluorescent aggregates with increasing membrane potential. An increase in red fluorescence intensity relative to green fluorescence intensity is an indication for membrane hyperpolarization.

### 4.10. Determination of Reactive Oxygen Species (ROS) Production

*G. vaginalis* (initial OD_600nm_ of 0.3) was centrifuged at 5000× *g* for 5 min and resuspended in 1 mL of BHI broth containing 0.2 mg/mL nitro blue tetrazolium (NBT) chloride (Cayman Chemical Company, Ann Arbor, MI, USA) and various concentrations of CBD (0–10 μg/mL) or 0.1% ethanol as a control. NBT is reduced by free oxygen radicals into an insoluble blue formazan [88]. The samples were incubated at 37 °C and after various time points the samples were centrifuged at 21,000× *g* for 3 min, the supernatant was discarded, the pellet containing the formazan precipitate was dissolved in 100 µL of DMSO by repeated pipetting, and the OD at 560 nm was immediately read in a Multiskan SkyHigh microplate reader. A fresh stock solution of 20 mg/mL NBT was prepared in DMSO prior to each experiment.

### 4.11. Live/Dead SYTO 9/PI Staining

Control and CBD-treated *G. vaginalis* were exposed to 3.3 μM SYTO 9 (Molecular Probes, Life Technologies, Carlsbad, CA, USA) and 10 μg/mL propidium iodide (PI) (Sigma, St. Louis, MO, USA) in 1 mL PBS for 20 min and then the green and red fluorescence intensities were measured by flow cytometry (LSR Fortessa flow cytometer) using the excitation/emission of 488 nm/520 nm for SYTO 9 and 561 nm/586 nm for PI [73]. SYTO 9 enters both live and dead bacteria and emits green fluorescence when bound to nucleic acids. PI is positively charged and can only penetrate bacteria with a perforated membrane. It emits red fluorescence when bound to nucleic acids.

### 4.12. Determination of the Bacterial DNA Content

Control and CBD-treated *G. vaginalis* were resuspended in 100 µL PBS and then fixed by dropwise addition of 1 mL methanol while vortexing the samples. After a one-hour incubation at minus 20, the bacteria were settled by centrifugation and rehydrated in 1 mL PBS. This was followed by a 30 min incubation with 1 µg/mL DAPI in PBS at room temperature and measuring the fluorescence intensities by flow cytometry using the excitation/emission of 405 nm/450 nm [73]. DAPI emits blue fluorescence when bound to DNA.

### 4.13. Membrane Staining with Nile Red

Control and CBD-treated *G. vaginalis* were incubated with 10 µg/mL Nile Red (APExBIO, Houston, TX, USA) during the last 30 min of incubation at 37 °C and the fluorescence intensities were measured using the excitation/emission of 561 nm/635 nm in the Fortezza flow cytometer [73]. Nile Red emits red fluorescence when integrated into the cell membrane.

### 4.14. Detection of Lipid Peroxidation

To determine whether CBD causes lipid peroxidation, we used the fluorescent lipid peroxidation sensor C11-Bodipy 581/591 undecanoic acid (Cayman Chemical Company, Ann Arbor, MI, USA). The oxidation of C11-Bodipy leads to a shift in its emission peak from red (around 590 nm) to green (around 510 nm) [58]. *G. vaginalis* was incubated with various concentrations of CBD and 10 µM C11-Bodipy for various durations (2 h, 4 h, and 8 h). At the end of incubation, the bacteria were washed in PBS and the green fluorescence intensity was determined by flow cytometry using the excitation/emission wavelengths of 488 nm/530 nm. In addition, we tested lipid peroxidation using the Lipid Peroxidation Assay Kit (A319696, Antibodies.com LLC, Cambridge, UK) according to the manufacturer’s instructions. This assay is based on the detection of malondialdehyde produced as a byproduct of lipid peroxidation. Arachidonic acid (Nu-Check Prep, 109 W Main St, Elysian, MN, USA), which is known to induce lipid peroxidation [57], was used as a positive control.

### 4.15. Quantitative Real-Time PCR

For gene expression studies, *G. vaginalis* with an initial OD_600nm_ of 0.25 in 10 mL of 50% BHI/50% Wilkins broth with 1% D-glucose was incubated in the absence or presence of 2.5 or 5 µg/mL CBD for 6 h at 37 °C under anaerobic conditions. The bacterial pellet was resuspended in 1 mL of RNA protect Bacterial Reagent (Qiagen, Hilden, Germany) and kept on ice for 5 min. Thereafter, the bacteria were recentrifuged, resuspended in 1 mL of Tri-Reagent (Sigma, St. Louis, MO, USA), and transferred to Type B bead tubes (Macherey–Nagel, Düren, Germany) [43]. The samples were then subjected to hard vortexing in a FastPrep FP120 BIO 101 Cell Disruption System (Savant) three times for 45 sec at a speed of 4.5 m/s. The samples were kept on ice between each pulse. The samples were then cleared from the beads by centrifugation, RNA was isolated according to the Tri-Reagent protocol, and the RNA pellet was washed twice with 70% ethanol. The integrity of the RNA was analyzed on a 1% agarose gel and the RNA concentration was determined by a NanoDrop microvolume spectrophotometer instrument. The RNA was reverse transcribed into cDNA using the AB high-capacity cDNA reverse transcription kit (Applied Biosystems, Vilnius, Lithuania), which uses random primers. Real-time PCR was performed using 10 ng of cDNA, 300 nM forward/reverse primer mixture (Supplementary Table S1) and Luna Universal qPCR Master Mix (New England BioLabs, Inc., Ipswich, MA, United States). The fold-change calculations was done using the 2^−ΔΔCt^ method, where an average was made from calculations obtained from the four reference genes *gyrA*, *gap*, *pgi*, and *rpoC* [85,89] and each treated sample was calculated against each control sample in triplicate.

### 4.16. Interaction of G. vaginalis with HeLa Cervical Epithelial Cells

The day before the experiment, 2 × 10^5^ HeLa cervical carcinoma cells (CCL-2, ATCC) were seeded in 2 mL of Dulbecco’s Modified Eagle Medium (DMEM; Sigma, St. Louis, MO, USA) supplemented with 8% heat-inactivated fetal calf serum (FCS) (Sigma, St. Louis, MO, USA), 2 mM L-glutamine, 1 mM sodium pyruvate, and antibiotics (penicillin–streptomycin) in 24-well culture plates, resulting in a monolayer after a 24 h incubation at 37 °C. The cells were then washed three times with 2 mL of RPMI supplemented with 1% heat-inactivated fetal calf serum (FCS) without antibiotics. After removing the medium, 1 mL of *G. vaginalis* at an initial OD_600nm_ of 0.25 was incubated on the HeLa monolayer in the absence or presence of various concentrations of CBD or 0.1% ethanol for 4 h at 37 °C under anaerobic conditions. At the end of incubation, the HeLa cells were washed three times with PBS and then the bacterial DNA was released by incubating the samples in 500 µL of 0.04 M NaOH at 60 °C for 1 h, followed by neutralization to pH 7.5 by the addition of 100 µL of 1 M Tris pH 6.8 [90]. The number of bacteria bound to the HeLa cells was determined according to the relative amount of bacterial DNA by quantitative real-time PCR with primers specific to the 16S rRNA of G. vaginalis (Supplementary Table S1). The amount of bacterial DNA was determined according to a standard curve obtained from purified *G. vaginalis* DNA. HeLa cells without bacteria served as negative controls and HeLa cells incubated with *G. vaginalis* in the absence of CBD served as positive controls.

### 4.17. Phagocytosis of CFSE-Labeled G. vaginalis by RAW 264.7 Macrophages

A total of 5 × 10^5^ RAW 264.7 macrophages (TIB-71, ATCC) were seeded in 6-well plates 2 days prior to the experiment in 2 mL of DMEM supplemented with 10% heat-inactivated FCS, 2 mM L-glutamine, 1 mM sodium pyruvate, and antibiotics (penicillin–streptomycin). On the day of the experiment, the macrophages were washed three times with 2 mL of RPMI supplemented with 1% heat-inactivated FCS without antibiotics and then 2 mL of carboxyfluorescein N-succinimidyl ester (CFSE)-labeled *G. vaginalis* at an initial OD_600nm_ of 0.1 was added to the washed macrophages for a 1 h incubation at 37 °C. At the end of incubation, the macrophages were washed twice with 2 mL of PBS, then scraped into 1 mL of PBS with a cell scraper, centrifuged at 5000× *g* for 5 min, resuspended in 1 mL of PBS, and filtrated through a 40 µm cell strainer (Falcon, Corning Science Mexico S.A. de C.V., Reynosa, Tamaulipas, Mexico). The green fluorescence intensity of the macrophages was read in a LSR Fortessa flow cytometer at excitation/emission wavelengths of 488 nm/530 nm. Macrophages without bacteria were used as negative controls for autofluorescence and CFSE-labeled bacteria were used as positive controls.

Green fluorescence-labeling of the bacteria was performed by incubating PBS-washed *G. vaginalis* with 6 mL of a 15 µM CFSE (Invitrogen, Thermo Fisher Scientific) solution in PBS for 20 min at room temperature. The excess CFSE was neutralized with an equal volume of heat-inactivated FCS. The neutralization was repeated by resuspending the bacterial pellet in 6 mL of FCS to which 20 mL of RPMI (Sigma) was added. Thereafter, the CFSE-labeled bacteria were washed twice in RPMI with 1% FCS (without antibiotics), before being used in the phagocytosis assay [91].

### 4.18. Analysis of the Protein Composition of Control and CBD-Treated G. vaginalis by LC-MS/MS Analyses

To determine changes in the bacterial protein profile, the same amounts of control and CBD-treated bacteria (2 OD units) were washed in PBS and subjected to extraction in 100 µL of non-reduced 4 × Laemelli sample buffer (Bio-Rad Laboratories, Inc., Hercules, CA, USA), followed by 15 min in an ice-cold ultrasonic water bath and incubation at 95 °C for 20 min. Fifteen µL was loaded on an 11% gel for silver staining and 15 µL on a 4–15% gradient gel (Mini-Protean TGX Stain-free gel, Bio-Rad Laboratories, Inc., USA) and the proteins were analyzed by the Smoler Proteomics Center at the Technion-Israel Institute of Technology, Israel [92]. The samples were digested with trypsin, analyzed by liquid chromatography and tandem mass spectrometry (LC-MS/MS) on a Q-Exactive HF instrument (Thermo Fisher Scientific, Waltham, MA, USA), and identified by Proteome Discoverer 2.4 software against the *G. vaginalis* UP000070687 proteome (UniProt database) and a decoy database to determine the false discovery rate. All the identified peptides were filtered with high confidence, top rank, mass accuracy, and score versus charge state. High-confidence peptides have passed the 1% false discovery rate (FDR) threshold, which is the estimated fraction of false positives in a list of peptides. Semi-quantitation was performed by calculating the peak area of each peptide.

### 4.19. Statistical Analysis

The experiments were performed in triplicates or quadruplicates, with at least three independent repeats. The data are presented as the average ± standard deviation of a representative experiment. Statistical significance was determined when the *p* value was less than 0.05 using the Student’s *t*-test and ANOVA in the Microsoft Excel 2013 program with post-hoc corrections.

## Figures and Tables

**Table 1:** Protein expression significantly altered after a 6 h incubation of *G. vaginalis* CI-1 with 2.5 and 5 µg/mL CBD. Twelve proteins were significantly downregulated and four proteins were significantly upregulated out of 386 proteins detected on LC-MS/MS analysis. The CBD-treated samples were compared to both ethanol-treated and control samples. Proteins affected at least two-fold by both CBD concentrations in comparison to both control and ethanol-treated samples are shown.

Accession	Description	Gene Symbol	MW [kDa]	Fold Change 2.5 µg/mL CBD	Fold Change 5 µg/mL CBD
A0A133P1J7	Large ribosomal subunit protein bL12 OS = Gardnerella vaginalis OX = 2702 GN = rplL PE = 3 SV = 1	*rplL*	14.1	0.54 ± 0.16	0.49 ± 0.15
A0A133NXW3	Small ribosomal subunit protein uS7 OS = Gardnerella vaginalis OX = 2702 GN = rpsG PE = 3 SV = 1	*rpsG*	17.6	0.40 ± 0.04	0.47 ± 0.05
A0A133NSL3	50S ribosomal protein L35 OS = Gardnerella vaginalis OX = 2702 GN = HMPREF3208_01112 PE = 3 SV = 1		6.7	0.43 ± 0.06	0.49 ± 0.07
A0A133NTN3	Ribosome-recycling factor OS = Gardnerella vaginalis OX = 2702 GN = frr PE = 3 SV = 1	*frr*	20.2	0.36 ± 0.01	0.39 ± 0.01
A0A133P2W2	Large ribosomal subunit protein bL34 OS = Gardnerella vaginalis OX = 2702 GN = rpmH PE = 3 SV = 1	*rpmH*	5.3	0.36 ± 0.01	0.31 ± 0.01
A0A133NSQ5	DUF3052 domain-containing protein OS = Gardnerella vaginalis OX = 2702 GN = HMPREF3208_01083 PE = 4 SV = 1		15.3	0.29 ± 0.02	0.37 ± 0.03
A0A133NTQ7	RNA polymerase-binding protein RbpA OS = Gardnerella vaginalis OX = 2702 GN = rbpA PE = 3 SV = 1	*rbpA*	13.3	0.32 ± 0.08	0.54 ± 0.15
A0A133P117	L-threonylcarbamoyladenylate synthase OS = Gardnerella vaginalis OX = 2702 GN = HMPREF3208_00379 PE = 3 SV = 1		24.3	0.45 ± 0.02	0.28 ± 0.012
A0A133P356	HAD hydrolase, family IA, variant 3 OS = Gardnerella vaginalis OX = 2702 GN = HMPREF3208_00068 PE = 4 SV = 1		26.8	0.32 ± 0.02	0.42 ± 0.03
A0A133NWM4	Adenylate kinase OS = Gardnerella vaginalis OX = 2702 GN = adk PE = 3 SV = 1	*adk*	20.7	0.48 ± 0.03	0.49 ± 0.03
A0A133NZS0	Single-stranded DNA-binding protein OS = Gardnerella vaginalis OX = 2702 GN = HMPREF3208_00467 PE = 3 SV = 1		20.9	0.35 ± 0.02	0.48 ± 0.02
A0A133NSC1	dUTPase OS = Gardnerella vaginalis OX = 2702 GN = HMPREF3208_01171 PE = 4 SV = 1		10.6	0.39 ± 0.06	0.47 ± 0.07
A0A133NZ97	Cysteine synthase OS = Gardnerella vaginalis OX = 2702 GN = HMPREF3208_00510 PE = 3 SV = 1		33	2.48 ± 0.56	2.43 ± 0.55
A0A133NWM1	Exonuclease VII, large subunit (Fragment) OS = Gardnerella vaginalis OX = 2702 GN = HMPREF3208_00772 PE = 4 SV = 1		20.1	2.08 ± 0.47	1.83 ± 0.42
A0A133P333	Beta sliding clamp OS = Gardnerella vaginalis OX = 2702 GN = HMPREF3208_00152 PE = 3 SV = 1		41.4	1.89 ± 0.17	2.05 ± 0.19
A0A133NWD1	Aldehyde-alcohol dehydrogenase OS = Gardnerella vaginalis OX = 2702 GN = HMPREF3208_00785 PE = 3 SV = 1		99	1.99 ± 0.46	1.72 ± 0.39

## Data Availability

All relevant data are presented in the figures, tables, and Supplementary Data. Raw data will be made available on reasonable request.

## Supplementary Materials

The following supporting information can be downloaded at: https://www.mdpi.com/article/10.3390/antibiotics14020136/s1, Supplementary Figure S1. Anti-bacterial effect of CBD on *G. vaginalis* CI-1 as determined by changes in optical densities. Supplementary Figure S2. Bactericidal effect of CBD on *G. vaginalis* CI-1 as determined by bacterial drop assay. Supplementary Figure S3. CBD treatment did not cause cytoplasmic leakage of the nucleic acid dye SYTO 9, and there is no appearance of a PI^high^ cell population. Supplementary Figure S4. CBD only slightly reduced the DNA content per bacterium after a 24 h incubation at the highest concentrations of 5 and 10 µg/mL. Supplementary Figure S5. HR-SEM images (×5000 magnifications) of control and CBD-treated *G. vaginalis* CI-1. Supplementary Figure S6. Nile red staining of *G. vaginalis* CI-1 that have been incubated with different concentrations of CBD for 4 h and 24 h. Supplementary Figure S7. CBD did not cause lipid peroxidation in *G. vaginalis*. Supplementary Figure S8. A. Effect of CBD on vaginolysin, transport-related and biofilm-related gene expression. Supplementary Figure S9. CBD did not prevent the phagocytosis of *G. vaginalis* by RAW 264.7 macrophages. Supplementary Figure S10. SDS-PAGE gel electrophoresis of protein extracts and real-time qPCR analysis of ribosome-related genes after a 6 h incubation. Supplementary Figure S11: Colonies of *Gardnerella vaginalis* CI-1 on chocolate blood agar plate. Supplementary Table S1; Certificate of Analysis of CBD.

## Abbreviations

The following abbreviations are used in this manuscript:

BV	bacterial vaginosis
CBD	cannabidiol
CFSE	carboxyfluorescein N-succinimidyl ester
HR-SEM	high-resolution scanning electron microscope
MTT	3-(4,5-dimethyl-2-thiazolyl)-2,5-diphenyl-2H-tetrazolium bromide
NBT	nitro blue tetrazolium
RFI	Relative Fluorescence Intensity
RLU	relative luminescence units
ROS	reactive oxygen species
