# Supplementary material for: Cannabidiol (CBD) Acts as an Antioxidant on Gardnerella vaginalis, Resulting in Reduced Metabolic Activity, Loss of Survivability, and Elimination of Biofilms
Source: Antibiotics (Basel). 2025 Feb 1;14(2):136. doi: 10.3390/antibiotics14020136 (PMC11851883; doi:10.3390/antibiotics14020136)
Supplement: Supplementary file 1 [file antibiotics-14-00136-s001.zip › antibiotics-3451154-supplementary.pptx]

## Slide 1
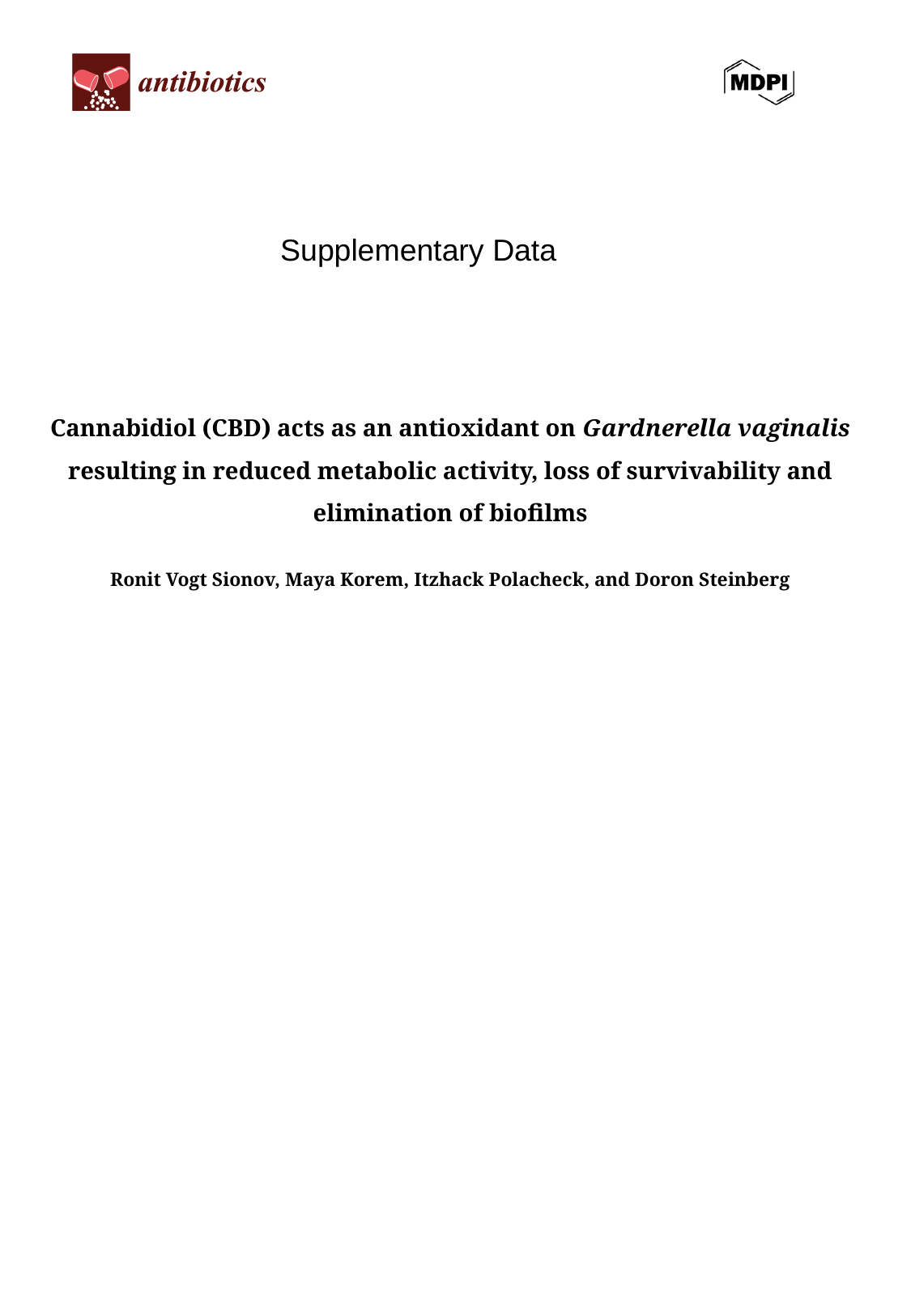

Supplementary Data
Cannabidiol (CBD) acts as an antioxidant on Gardnerella vaginalis resulting in reduced metabolic activity, loss of survivability and elimination of biofilms
Ronit Vogt Sionov, Maya Korem, Itzhack Polacheck, and Doron Steinberg

## Slide 2
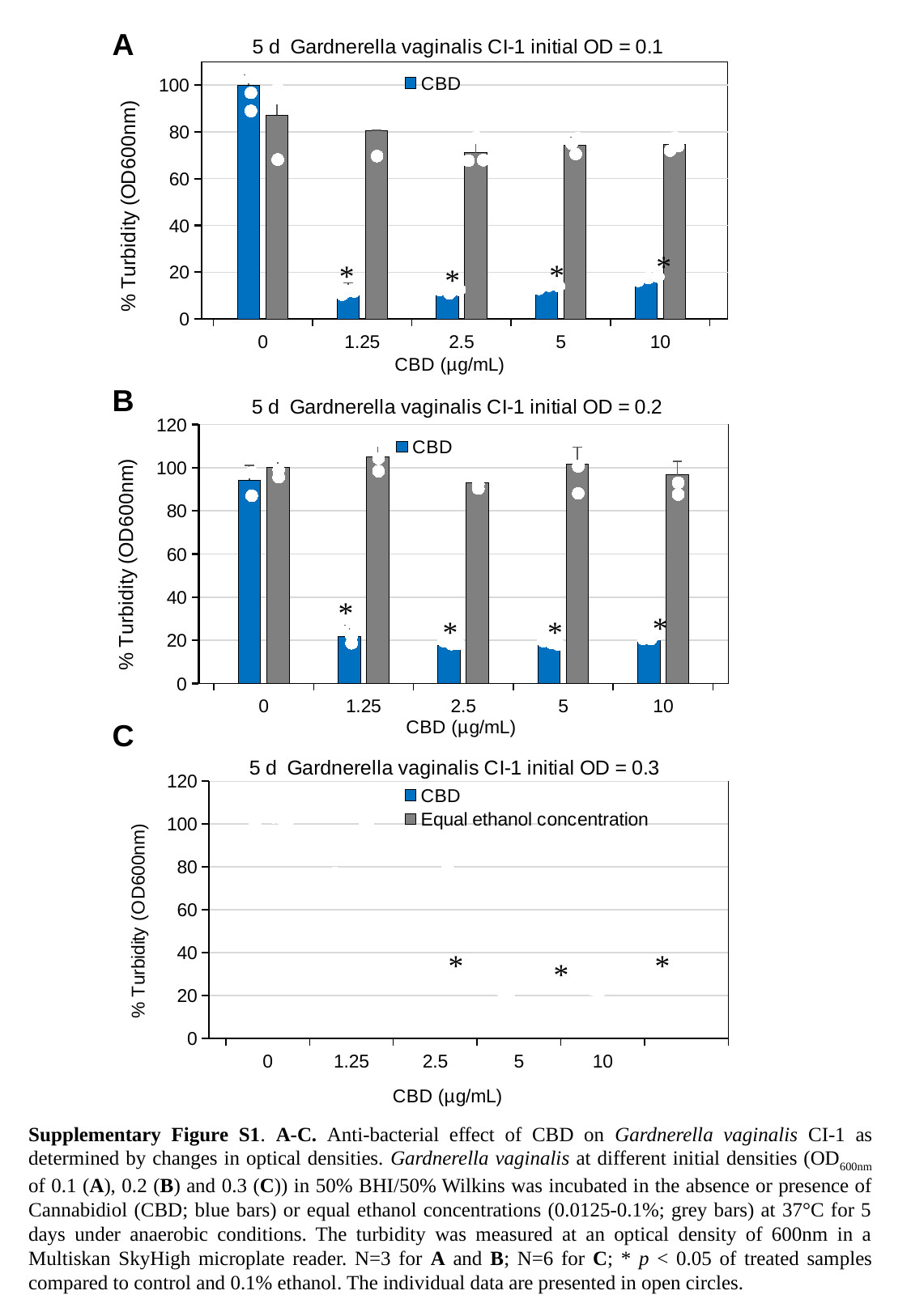

[unsupported chart]
*
*
*
*
A
B
[unsupported chart]
*
*
*
*
C
### Chart: 5 d Gardnerella vaginalis CI-1 initial OD = 0.3
| Category | | Equal ethanol concentration | | | | | | | | | | |
|---|---|---|---|---|---|---|---|---|---|---|---|---|
| 0 | 100.0 | 100.75922938217707 | 111.94837240201194 | 96.91563063490555 | 94.97959571035398 | 87.00768719749455 | 24.94068520451742 | 89.6270285660055 | 21.979690614026758 | 89.6270285660055 | 28.584986238967446 | 99.99050963272279 |
| 1.25 | 75.04982442820537 | 98.41510866470534 | 94.75182689570087 | 103.17927303786657 | 77.21362816741008 | 98.51001233747748 | 24.48514757521115 | 86.55214956818828 | 20.15754009680174 | 88.14653127076019 | 23.57407231659865 | 93.49909841510865 |
| 2.5 | 25.339280630160378 | 91.62000569422037 | 90.99364145392424 | 99.53497200341653 | 65.02799658346777 | 94.63794248837429 | 28.357217424314314 | 79.26354749928821 | 23.118534687292396 | 89.6270285660055 | 24.48514757521115 | 89.51314415867893 |
| 5 | 22.055613552244477 | 89.75989370788649 | 96.91563063490555 | 102.83761981588688 | 63.43361488089588 | 103.4070418525197 | 26.193413685109608 | 92.4741387491696 | 23.118534687292396 | 89.05760652937269 | 21.06861535541426 | 98.51001233747748 |
| 10 | 24.181455822340318 | 98.5289930720319 | 103.17927303786657 | 102.04042896460092 | 74.59428679889912 | 105.68472999905096 | 24.029609945904898 | 92.13248552718989 | 22.662997057986146 | 76.188668501471 | 22.890765872639264 | 101.12935370598844 |*
*
*
Supplementary Figure S1. A-C. Anti-bacterial effect of CBD on Gardnerella vaginalis CI-1 as determined by changes in optical densities. Gardnerella vaginalis at different initial densities (OD600nm of 0.1 (A), 0.2 (B) and 0.3 (C)) in 50% BHI/50% Wilkins was incubated in the absence or presence of Cannabidiol (CBD; blue bars) or equal ethanol concentrations (0.0125-0.1%; grey bars) at 37°C for 5 days under anaerobic conditions. The turbidity was measured at an optical density of 600nm in a Multiskan SkyHigh microplate reader. N=3 for A and B; N=6 for C; * p < 0.05 of treated samples compared to control and 0.1% ethanol. The individual data are presented in open circles.

## Slide 3
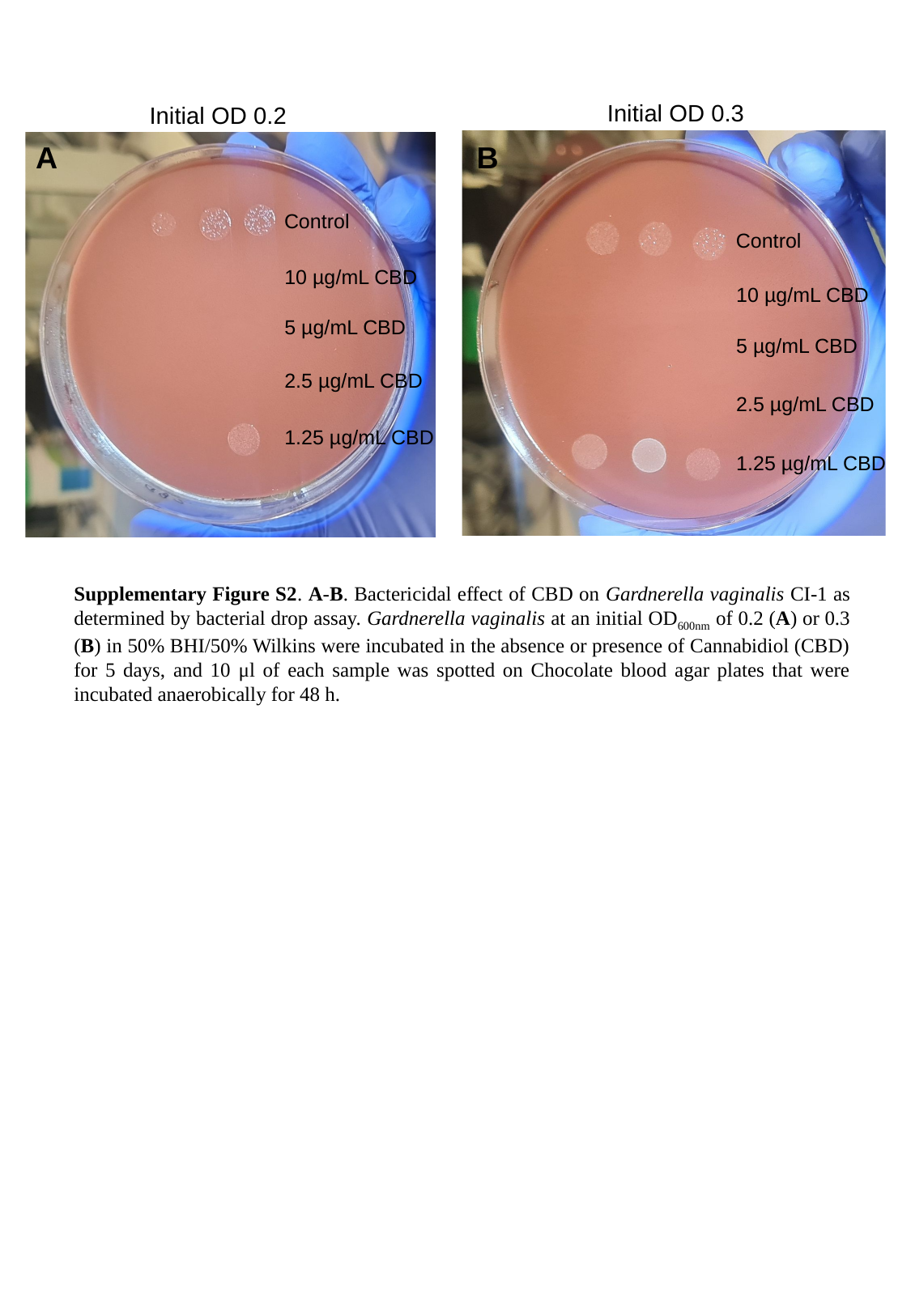

Initial OD 0.3
Initial OD 0.2
Control
Control
10 µg/mL CBD
10 µg/mL CBD
5 µg/mL CBD
5 µg/mL CBD
2.5 µg/mL CBD
2.5 µg/mL CBD
1.25 µg/mL CBD
1.25 µg/mL CBD
A
B
Supplementary Figure S2. A-B. Bactericidal effect of CBD on Gardnerella vaginalis CI-1 as determined by bacterial drop assay. Gardnerella vaginalis at an initial OD600nm of 0.2 (A) or 0.3 (B) in 50% BHI/50% Wilkins were incubated in the absence or presence of Cannabidiol (CBD) for 5 days, and 10 μl of each sample was spotted on Chocolate blood agar plates that were incubated anaerobically for 48 h.

## Slide 4
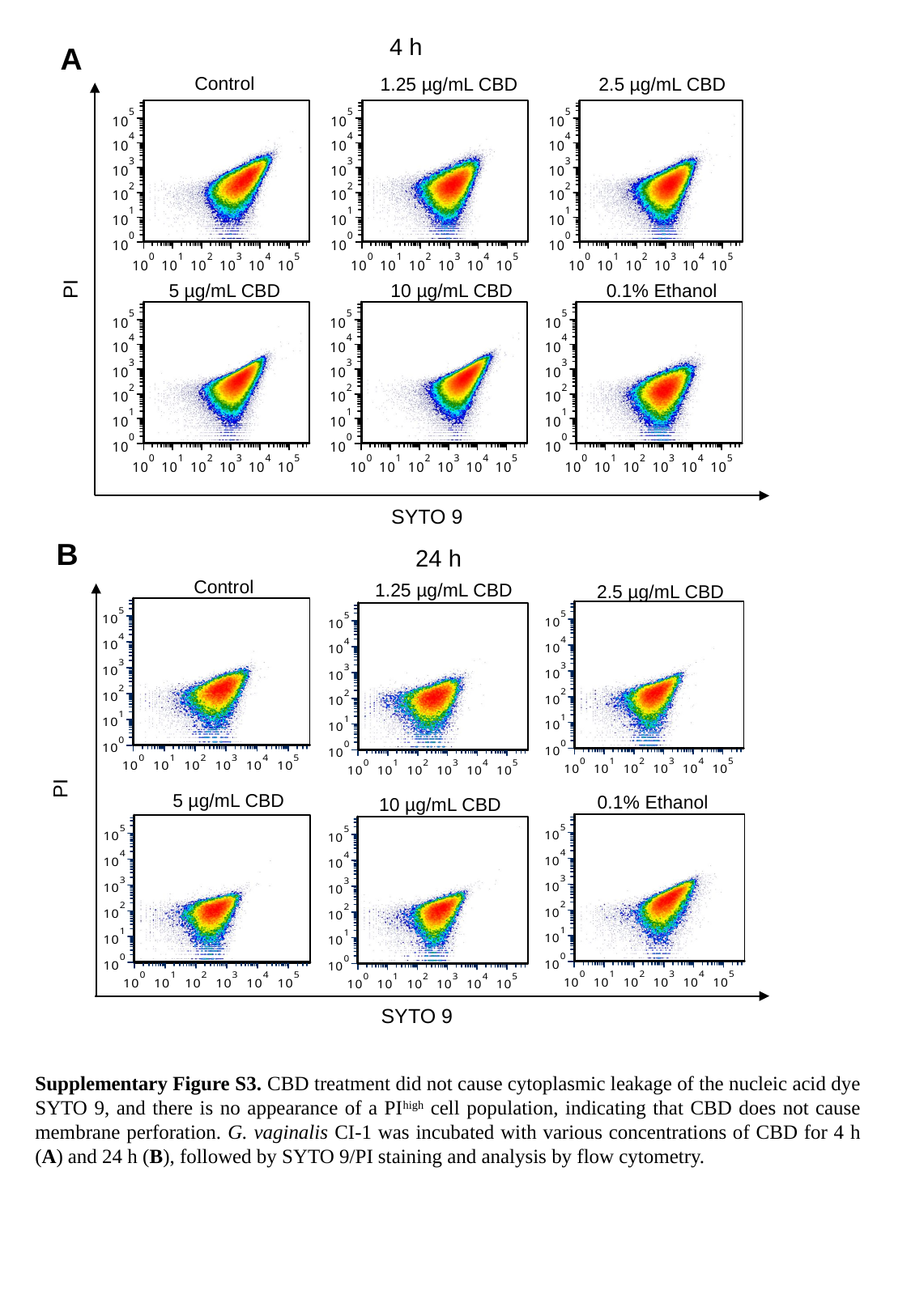

4 h
A
Control
1.25 µg/mL CBD
2.5 µg/mL CBD
PI
5 µg/mL CBD
10 µg/mL CBD
0.1% Ethanol
SYTO 9
B
24 h
Control
1.25 µg/mL CBD
2.5 µg/mL CBD
5 µg/mL CBD
0.1% Ethanol
10 µg/mL CBD
SYTO 9
PI
Supplementary Figure S3. CBD treatment did not cause cytoplasmic leakage of the nucleic acid dye SYTO 9, and there is no appearance of a PIhigh cell population, indicating that CBD does not cause membrane perforation. G. vaginalis CI-1 was incubated with various concentrations of CBD for 4 h (A) and 24 h (B), followed by SYTO 9/PI staining and analysis by flow cytometry.

## Slide 5
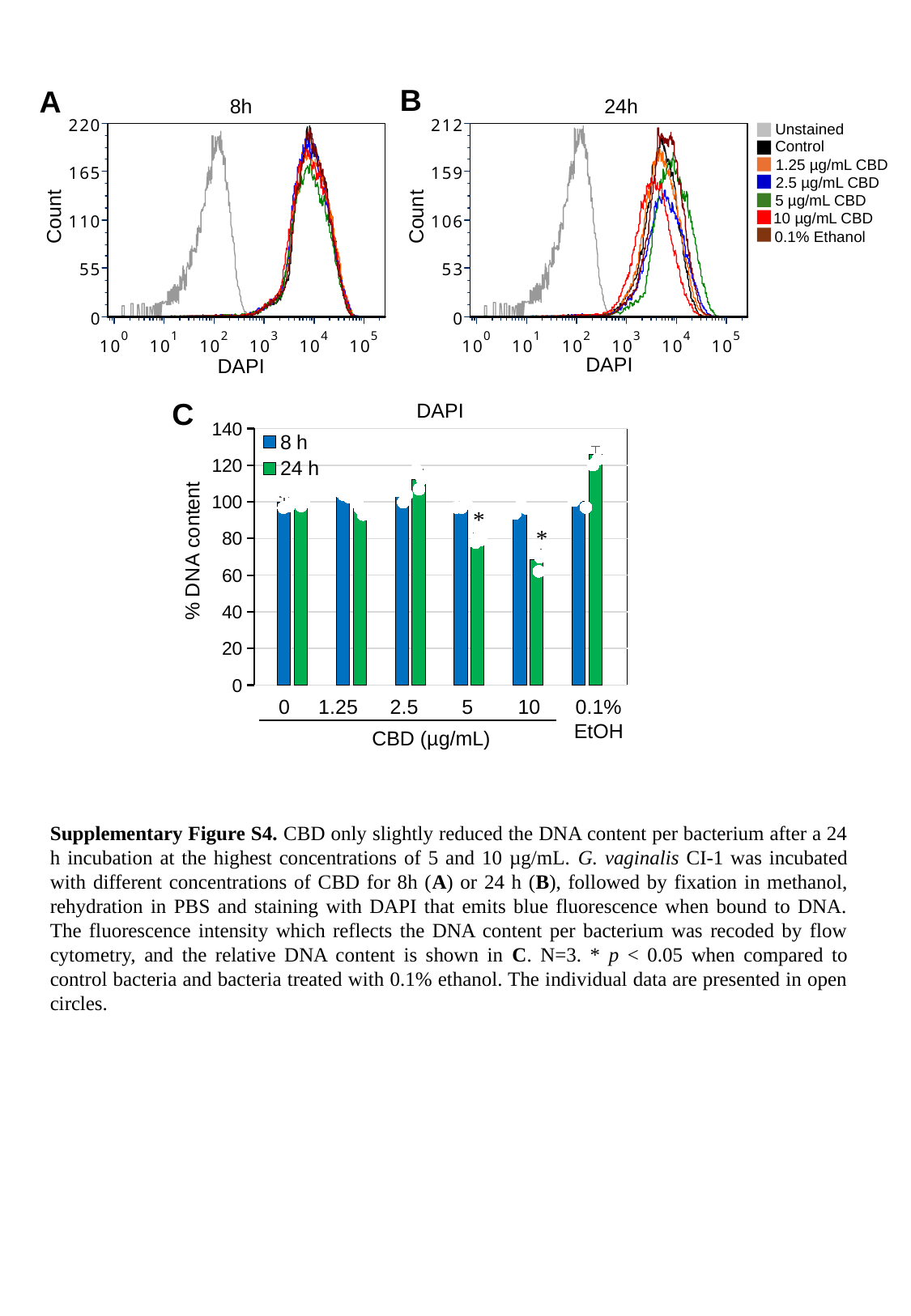

B
A
8h
24h
Unstained
Control
1.25 µg/mL CBD
2.5 µg/mL CBD
5 µg/mL CBD
10 µg/mL CBD
0.1% Ethanol
Count
Count
DAPI
DAPI
C
DAPI
[unsupported chart]
*
*
0
1.25
2.5
5
10
0.1%
EtOH
CBD (µg/mL)
Supplementary Figure S4. CBD only slightly reduced the DNA content per bacterium after a 24 h incubation at the highest concentrations of 5 and 10 µg/mL. G. vaginalis CI-1 was incubated with different concentrations of CBD for 8h (A) or 24 h (B), followed by fixation in methanol, rehydration in PBS and staining with DAPI that emits blue fluorescence when bound to DNA. The fluorescence intensity which reflects the DNA content per bacterium was recoded by flow cytometry, and the relative DNA content is shown in C. N=3. * p < 0.05 when compared to control bacteria and bacteria treated with 0.1% ethanol. The individual data are presented in open circles.

## Slide 6
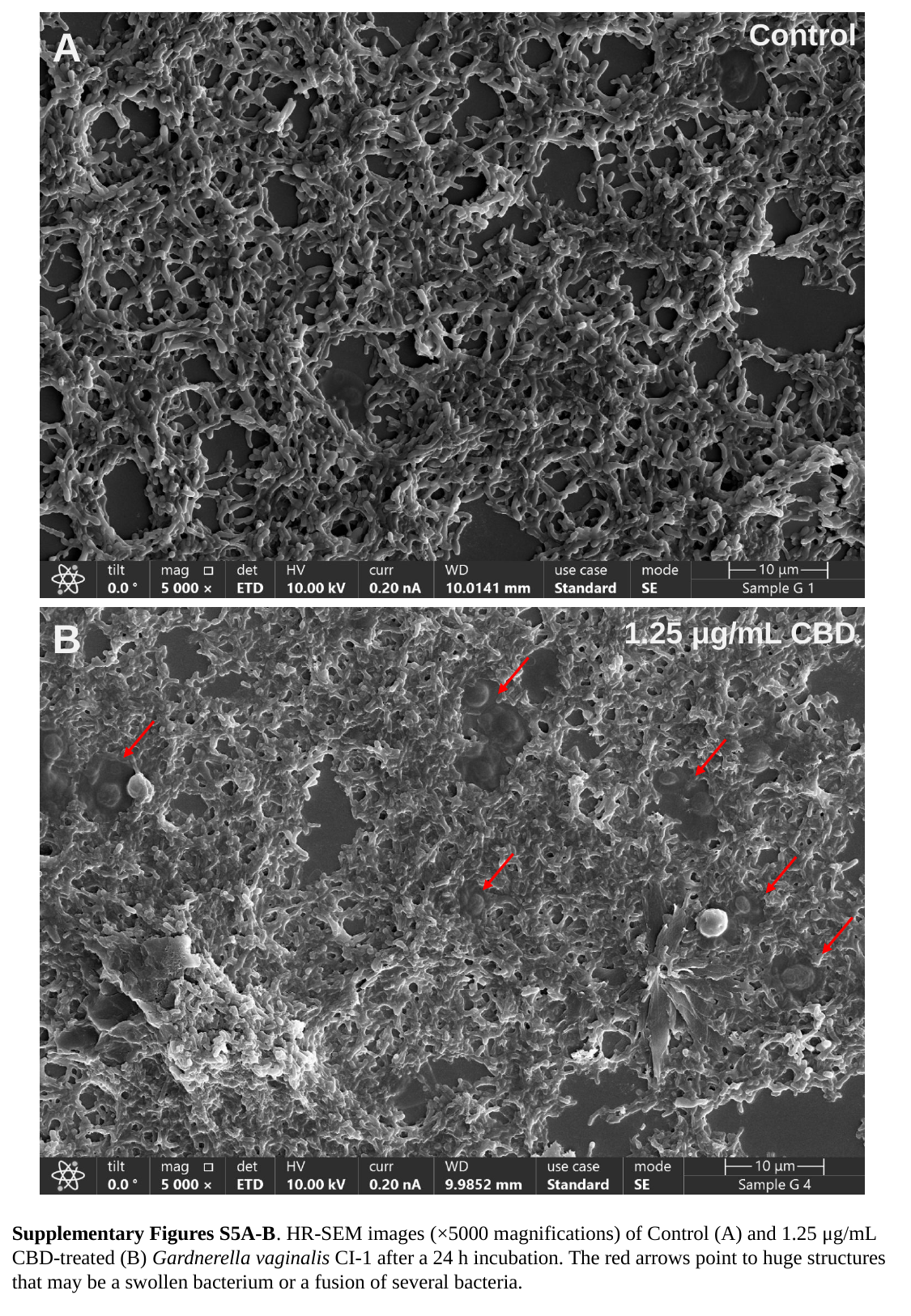

Control
A
1.25 μg/mL CBD
B
Supplementary Figures S5A-B. HR-SEM images (×5000 magnifications) of Control (A) and 1.25 μg/mL CBD-treated (B) Gardnerella vaginalis CI-1 after a 24 h incubation. The red arrows point to huge structures that may be a swollen bacterium or a fusion of several bacteria.

## Slide 7
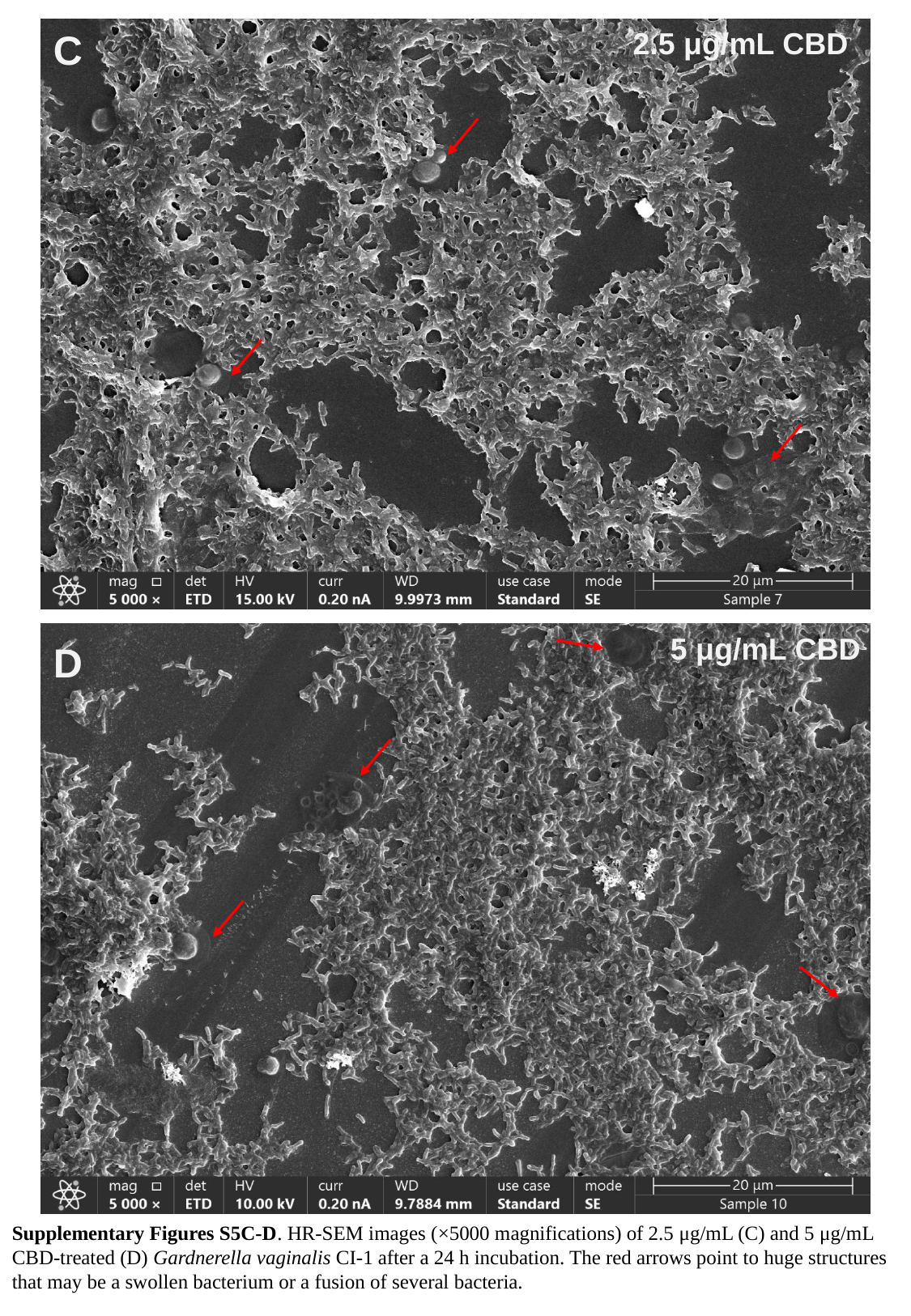

2.5 μg/mL CBD
C
5 μg/mL CBD
D
Supplementary Figures S5C-D. HR-SEM images (×5000 magnifications) of 2.5 μg/mL (C) and 5 μg/mL CBD-treated (D) Gardnerella vaginalis CI-1 after a 24 h incubation. The red arrows point to huge structures that may be a swollen bacterium or a fusion of several bacteria.

## Slide 8
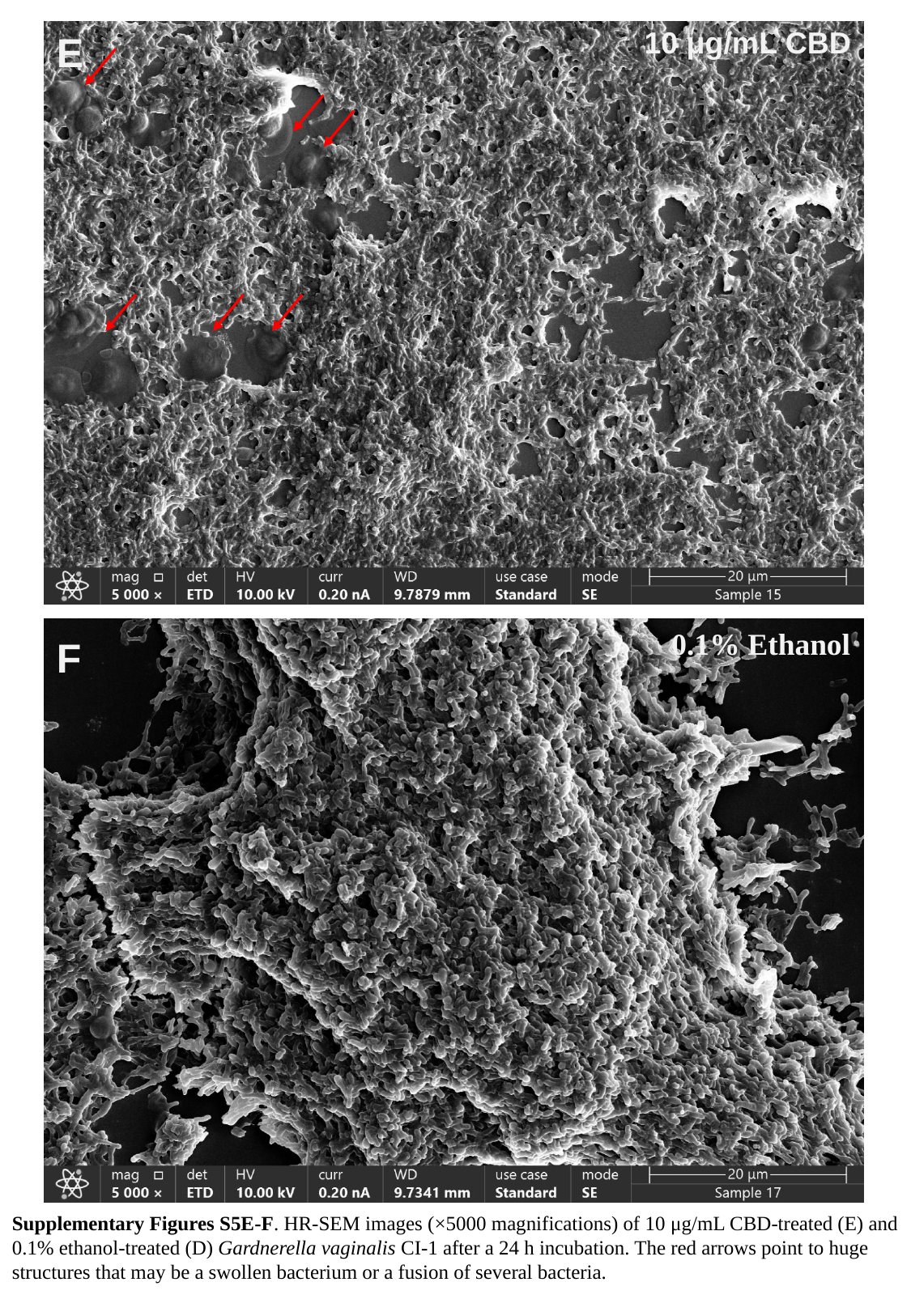

10 μg/mL CBD
E
0.1% Ethanol
F
Supplementary Figures S5E-F. HR-SEM images (×5000 magnifications) of 10 μg/mL CBD-treated (E) and 0.1% ethanol-treated (D) Gardnerella vaginalis CI-1 after a 24 h incubation. The red arrows point to huge structures that may be a swollen bacterium or a fusion of several bacteria.

## Slide 9
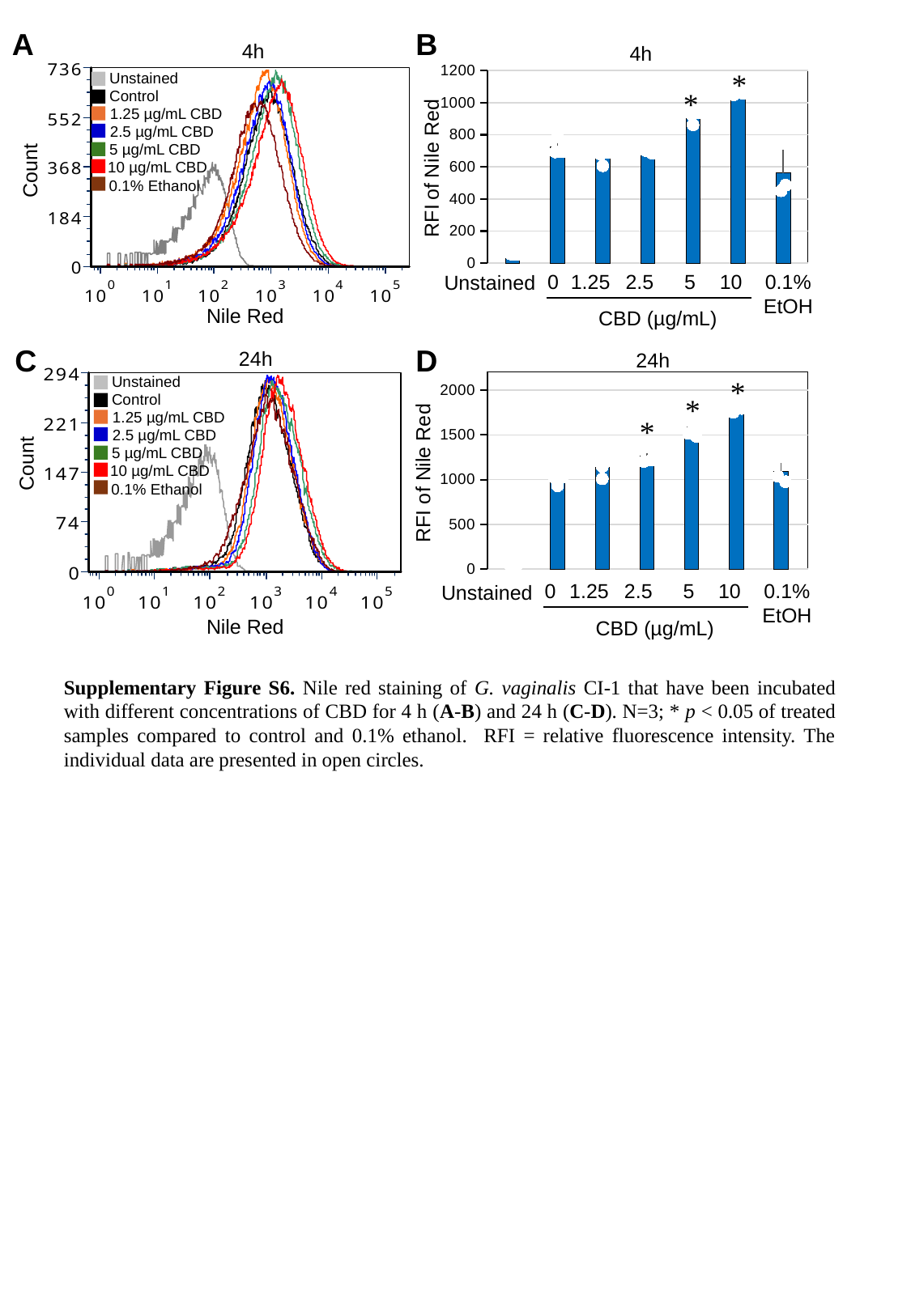

A
B
4h
4h
[unsupported chart]
Count
Nile Red
*
Unstained
Control
1.25 µg/mL CBD
2.5 µg/mL CBD
5 µg/mL CBD
10 µg/mL CBD
0.1% Ethanol
*
0
1.25
2.5
5
10
0.1%
EtOH
CBD (µg/mL)
Unstained
C
D
24h
24h
[unsupported chart]
Unstained
Control
1.25 µg/mL CBD
2.5 µg/mL CBD
5 µg/mL CBD
10 µg/mL CBD
0.1% Ethanol
*
*
*
Count
RFI of Nile Red
0
1.25
2.5
5
10
0.1%
EtOH
CBD (µg/mL)
Unstained
Nile Red
Supplementary Figure S6. Nile red staining of G. vaginalis CI-1 that have been incubated with different concentrations of CBD for 4 h (A-B) and 24 h (C-D). N=3; * p < 0.05 of treated samples compared to control and 0.1% ethanol. RFI = relative fluorescence intensity. The individual data are presented in open circles.

## Slide 10
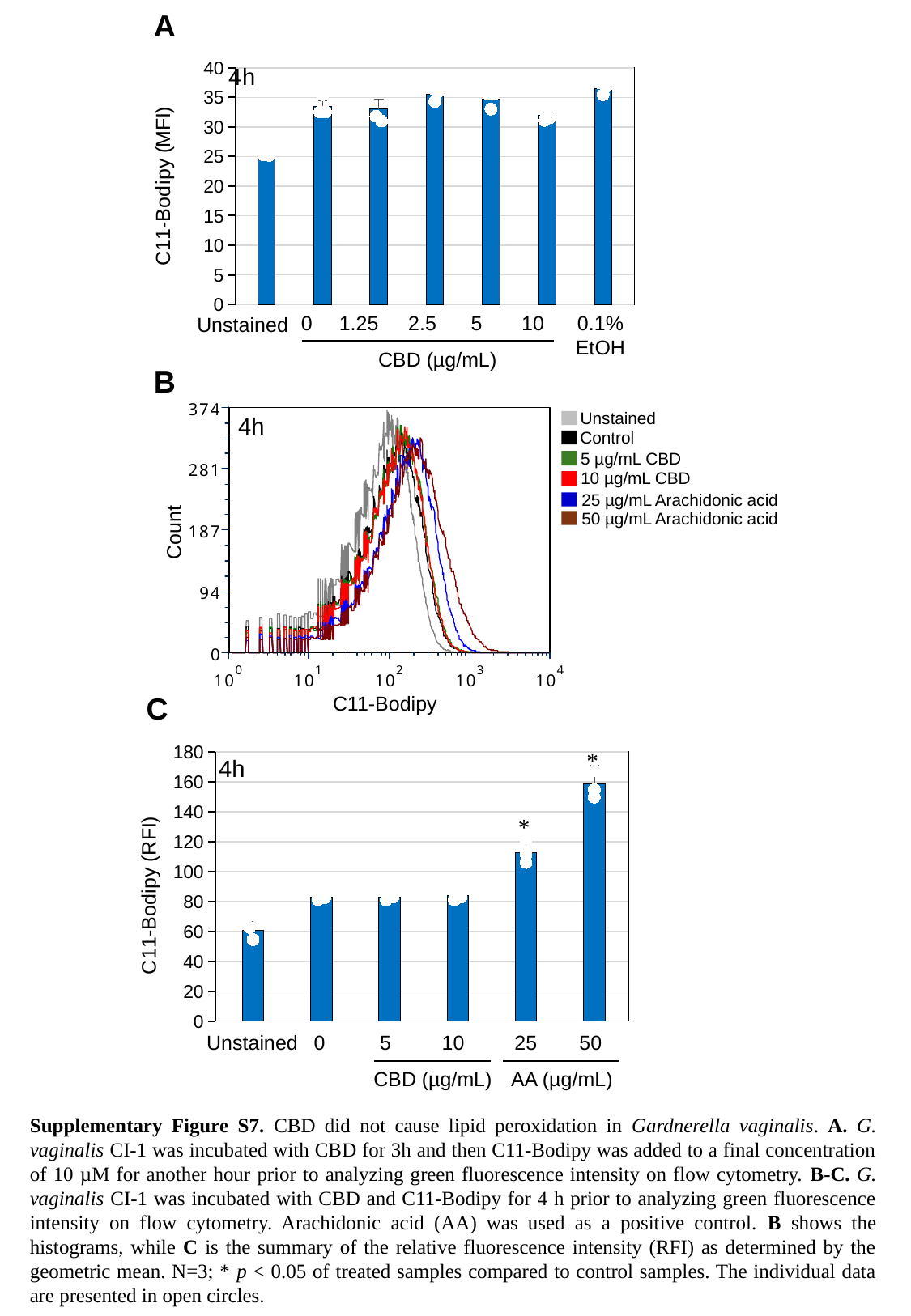

[unsupported chart]
A
0
1.25
2.5
5
10
0.1%
EtOH
CBD (µg/mL)
Unstained
B
Unstained
Control
5 µg/mL CBD
10 µg/mL CBD
25 µg/mL Arachidonic acid
50 µg/mL Arachidonic acid
4h
Count
C
[unsupported chart]
*
4h
*
Unstained
0
5
10
25
50
AA (µg/mL)
CBD (µg/mL)
C11-Bodipy
Supplementary Figure S7. CBD did not cause lipid peroxidation in Gardnerella vaginalis. A. G. vaginalis CI-1 was incubated with CBD for 3h and then C11-Bodipy was added to a final concentration of 10 µM for another hour prior to analyzing green fluorescence intensity on flow cytometry. B-C. G. vaginalis CI-1 was incubated with CBD and C11-Bodipy for 4 h prior to analyzing green fluorescence intensity on flow cytometry. Arachidonic acid (AA) was used as a positive control. B shows the histograms, while C is the summary of the relative fluorescence intensity (RFI) as determined by the geometric mean. N=3; * p < 0.05 of treated samples compared to control samples. The individual data are presented in open circles.

## Slide 11
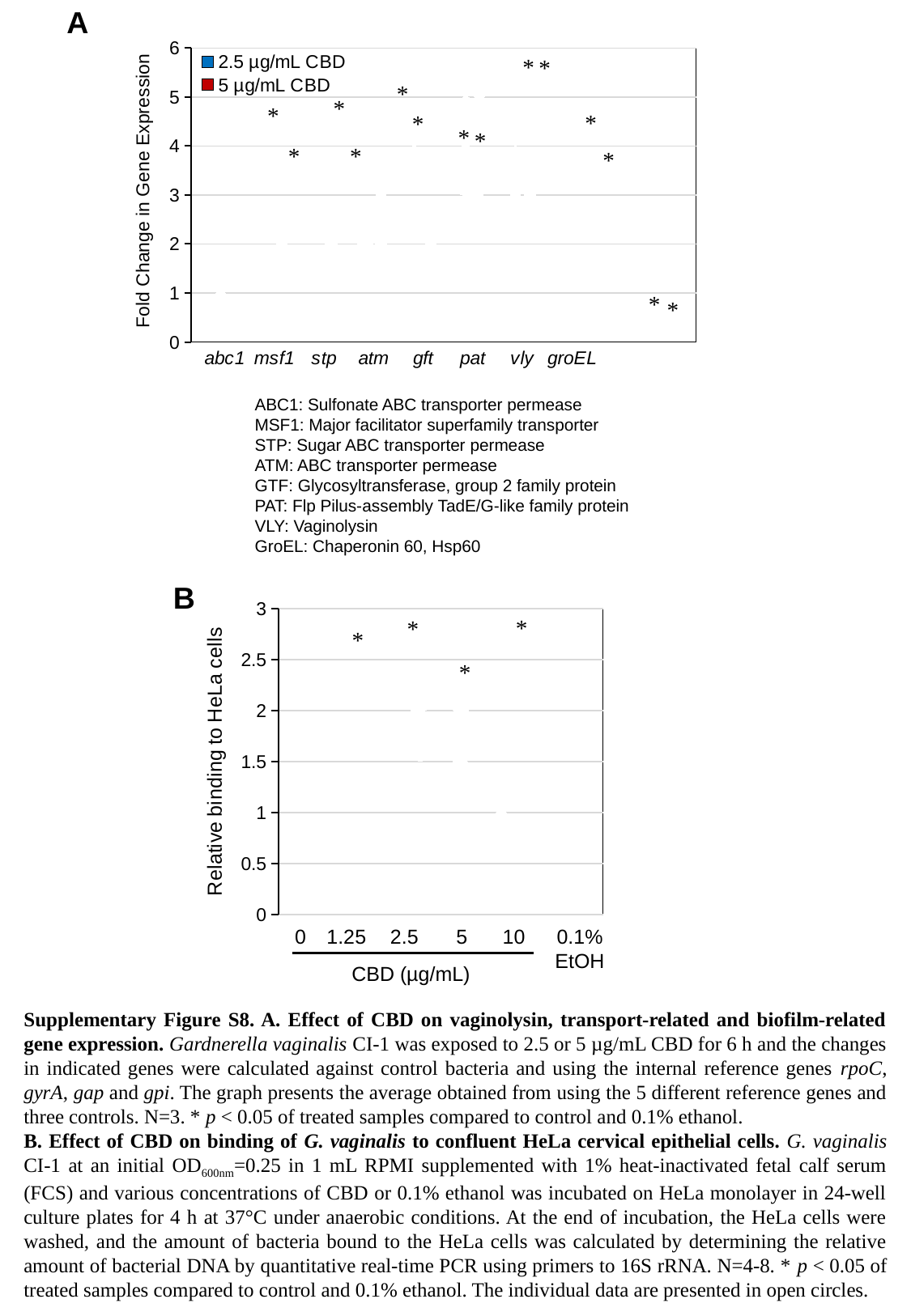

A
[unsupported chart]
*
*
*
*
*
*
*
*
*
*
*
*
Fold Change in Gene Expression
*
*
ABC1: Sulfonate ABC transporter permease
MSF1: Major facilitator superfamily transporter
STP: Sugar ABC transporter permease
ATM: ABC transporter permease
GTF: Glycosyltransferase, group 2 family protein
PAT: Flp Pilus-assembly TadE/G-like family protein
VLY: Vaginolysin
GroEL: Chaperonin 60, Hsp60
B
[unsupported chart]
*
*
*
*
0
1.25
2.5
5
10
0.1%
EtOH
CBD (µg/mL)
Supplementary Figure S8. A. Effect of CBD on vaginolysin, transport-related and biofilm-related gene expression. Gardnerella vaginalis CI-1 was exposed to 2.5 or 5 µg/mL CBD for 6 h and the changes in indicated genes were calculated against control bacteria and using the internal reference genes rpoC, gyrA, gap and gpi. The graph presents the average obtained from using the 5 different reference genes and three controls. N=3. * p < 0.05 of treated samples compared to control and 0.1% ethanol.
B. Effect of CBD on binding of G. vaginalis to confluent HeLa cervical epithelial cells. G. vaginalis CI-1 at an initial OD600nm=0.25 in 1 mL RPMI supplemented with 1% heat-inactivated fetal calf serum (FCS) and various concentrations of CBD or 0.1% ethanol was incubated on HeLa monolayer in 24-well culture plates for 4 h at 37°C under anaerobic conditions. At the end of incubation, the HeLa cells were washed, and the amount of bacteria bound to the HeLa cells was calculated by determining the relative amount of bacterial DNA by quantitative real-time PCR using primers to 16S rRNA. N=4-8. * p < 0.05 of treated samples compared to control and 0.1% ethanol. The individual data are presented in open circles.

## Slide 12
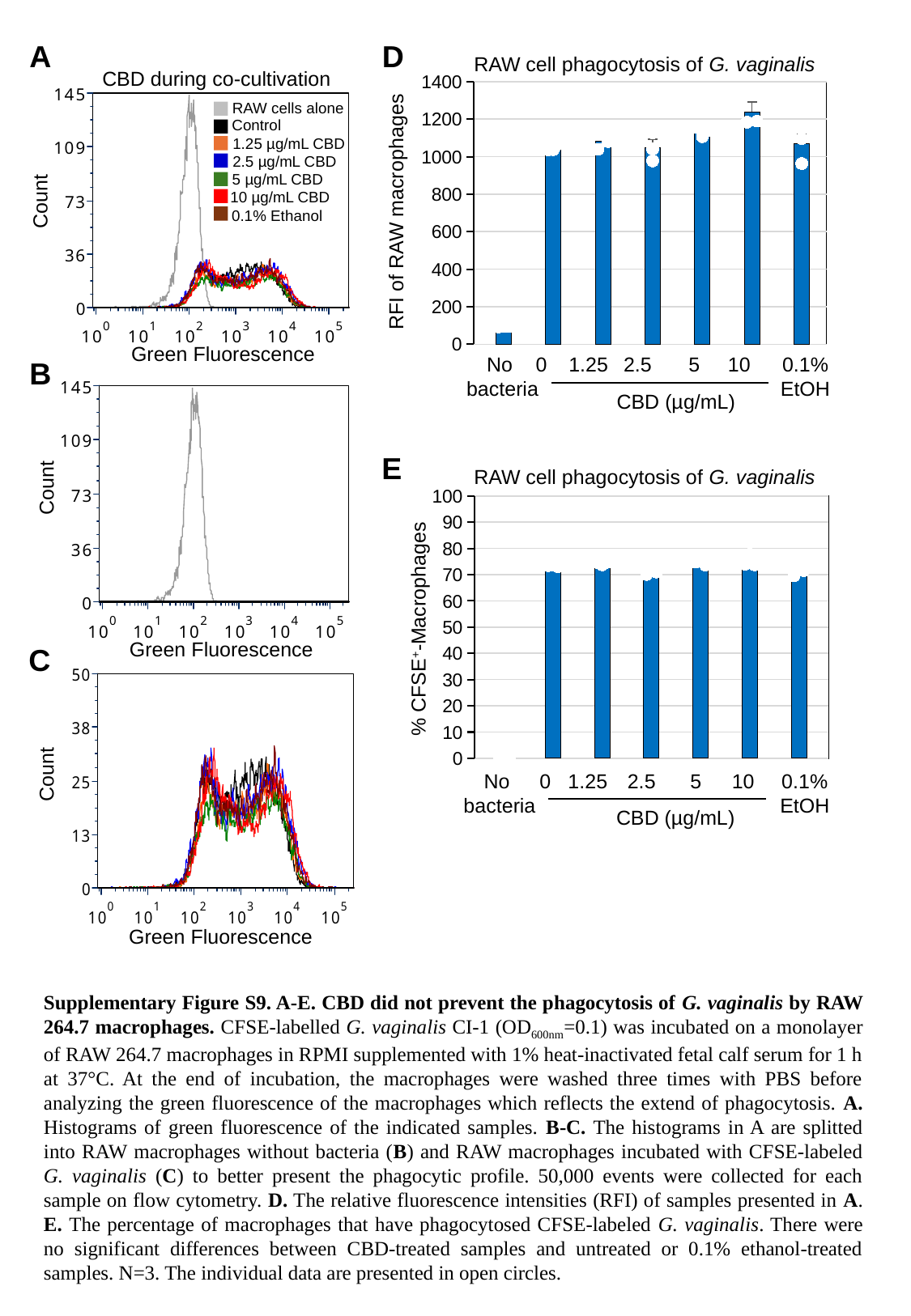

A
D
[unsupported chart]
RAW cell phagocytosis of G. vaginalis
CBD during co-cultivation
RAW cells alone
Control
1.25 µg/mL CBD
2.5 µg/mL CBD
5 µg/mL CBD
10 µg/mL CBD
0.1% Ethanol
Count
Green Fluorescence
Count
Green Fluorescence
Count
Green Fluorescence
RFI of RAW macrophages
0
1.25
2.5
5
10
0.1%
EtOH
CBD (µg/mL)
No
bacteria
B
E
RAW cell phagocytosis of G. vaginalis
[unsupported chart]
% CFSE+-Macrophages
C
0
1.25
2.5
5
10
0.1%
EtOH
CBD (µg/mL)
No
bacteria
Supplementary Figure S9. A-E. CBD did not prevent the phagocytosis of G. vaginalis by RAW 264.7 macrophages. CFSE-labelled G. vaginalis CI-1 (OD600nm=0.1) was incubated on a monolayer of RAW 264.7 macrophages in RPMI supplemented with 1% heat-inactivated fetal calf serum for 1 h at 37°C. At the end of incubation, the macrophages were washed three times with PBS before analyzing the green fluorescence of the macrophages which reflects the extend of phagocytosis. A. Histograms of green fluorescence of the indicated samples. B-C. The histograms in A are splitted into RAW macrophages without bacteria (B) and RAW macrophages incubated with CFSE-labeled G. vaginalis (C) to better present the phagocytic profile. 50,000 events were collected for each sample on flow cytometry. D. The relative fluorescence intensities (RFI) of samples presented in A. E. The percentage of macrophages that have phagocytosed CFSE-labeled G. vaginalis. There were no significant differences between CBD-treated samples and untreated or 0.1% ethanol-treated samples. N=3. The individual data are presented in open circles.

## Slide 13
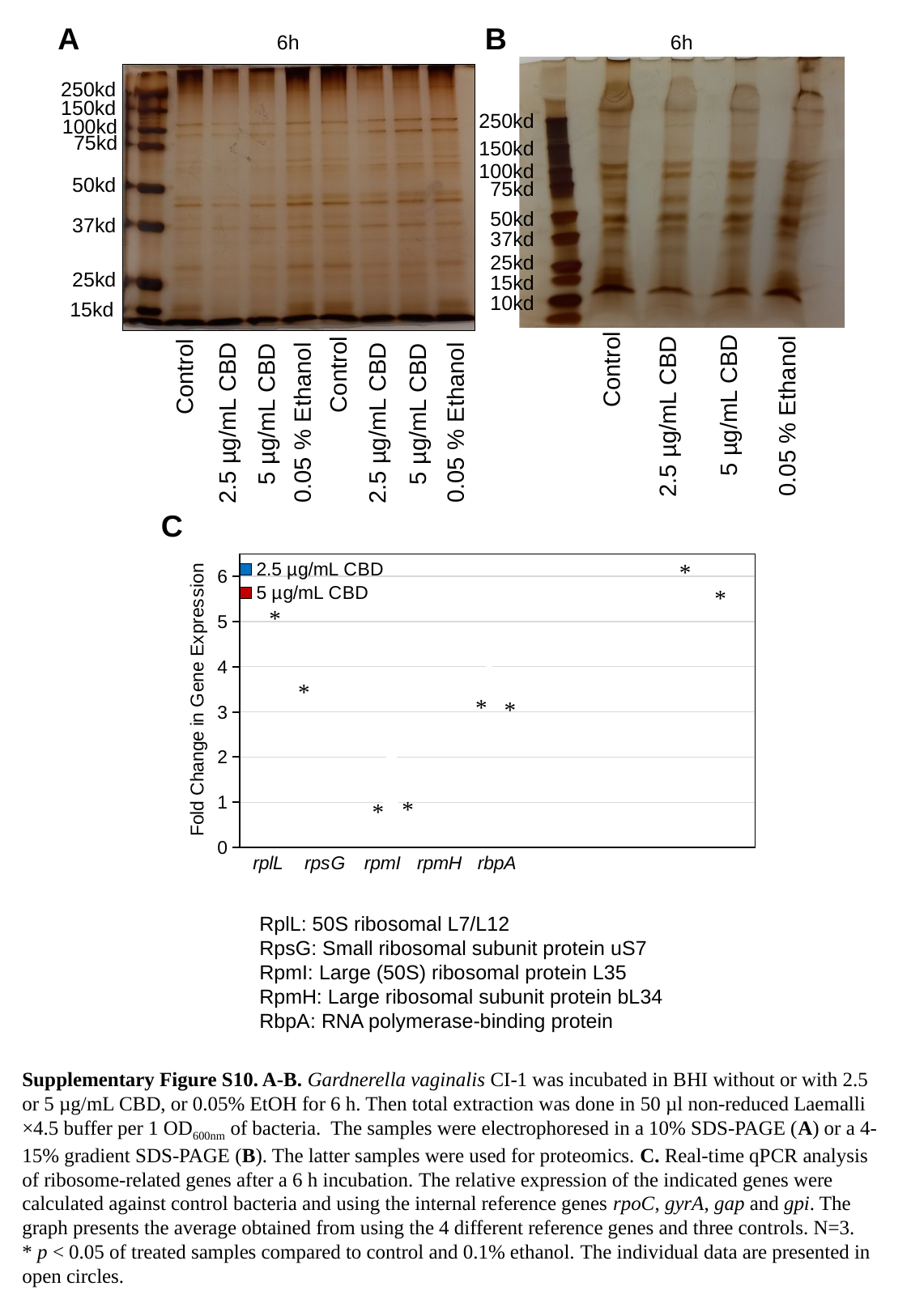

B
A
6h
6h
250kd
150kd
100kd
75kd
50kd
37kd
25kd
15kd
10kd
250kd
150kd
100kd
75kd
50kd
37kd
25kd
15kd
Control
Control
Control
5 µg/mL CBD
5 µg/mL CBD
5 µg/mL CBD
0.05 % Ethanol
2.5 µg/mL CBD
0.05 % Ethanol
0.05 % Ethanol
2.5 µg/mL CBD
2.5 µg/mL CBD
C
### Chart
| Category | | | | | | | | | | | | |
|---|---|---|---|---|---|---|---|---|---|---|---|---|
| rplL | 2.654332560850844 | 2.0586225098041675 | 2.3815805912289925 | 1.2743319402365025 | 0.2650395326236233 | 0.1751292032103223 | 1.6215727838661007 | 0.9952525640111292 | 0.6798794307401612 | 0.5367444682864072 | 3.240803455808704 | 2.692345706595238 |
| rpsG | 0.2950639654276389 | 0.35031059685242216 | 1.491407092281058 | 1.8034455364219226 | 0.22781420728565677 | 0.24784435660316873 | 1.676868906174427 | 1.4084899997446767 | 0.7639457691847743 | 0.7596053939844204 | 4.129512516441983 | 3.810230830565841 |
| rpmI | 1.9904409440434656 | 1.6979514068034898 | 2.2300437992707858 | 2.8358557939008424 | 0.3504611473920858 | 0.4566616146231924 | 2.3028133500017556 | 2.4508154858124778 | 0.5514599072151329 | 1.1839424679359638 | 2.324778243264545 | 4.402879044802576 |
| rpmH | 0.8216870474145235 | 0.9295917712024566 | 3.276644296023394 | 2.4963525808494054 | 0.24718310699035678 | 0.586275745491605 | 1.5843281484034104 | 2.386852556327102 | 1.2435791821516569 | 1.4890689647821296 | 4.613765578393114 | 5.1944592605554245 |
| rbpA | 3.638564400511895 | 3.419432678634495 | 2.1552373259732382 | 1.568096066683041 | 0.4268494259392003 | 0.430260572239322 | 2.7666215317716345 | 2.0820526806280855 | 0.7459280575902684 | 1.144205608900838 | 3.2266648770020554 | 4.469189711974614 |*
*
*
*
*
*
*
*
RplL: 50S ribosomal L7/L12
RpsG: Small ribosomal subunit protein uS7
RpmI: Large (50S) ribosomal protein L35
RpmH: Large ribosomal subunit protein bL34
RbpA: RNA polymerase-binding protein
Supplementary Figure S10. A-B. Gardnerella vaginalis CI-1 was incubated in BHI without or with 2.5 or 5 µg/mL CBD, or 0.05% EtOH for 6 h. Then total extraction was done in 50 µl non-reduced Laemalli ×4.5 buffer per 1 OD600nm of bacteria. The samples were electrophoresed in a 10% SDS-PAGE (A) or a 4-15% gradient SDS-PAGE (B). The latter samples were used for proteomics. C. Real-time qPCR analysis of ribosome-related genes after a 6 h incubation. The relative expression of the indicated genes were calculated against control bacteria and using the internal reference genes rpoC, gyrA, gap and gpi. The graph presents the average obtained from using the 4 different reference genes and three controls. N=3.
* p < 0.05 of treated samples compared to control and 0.1% ethanol. The individual data are presented in open circles.

## Slide 14
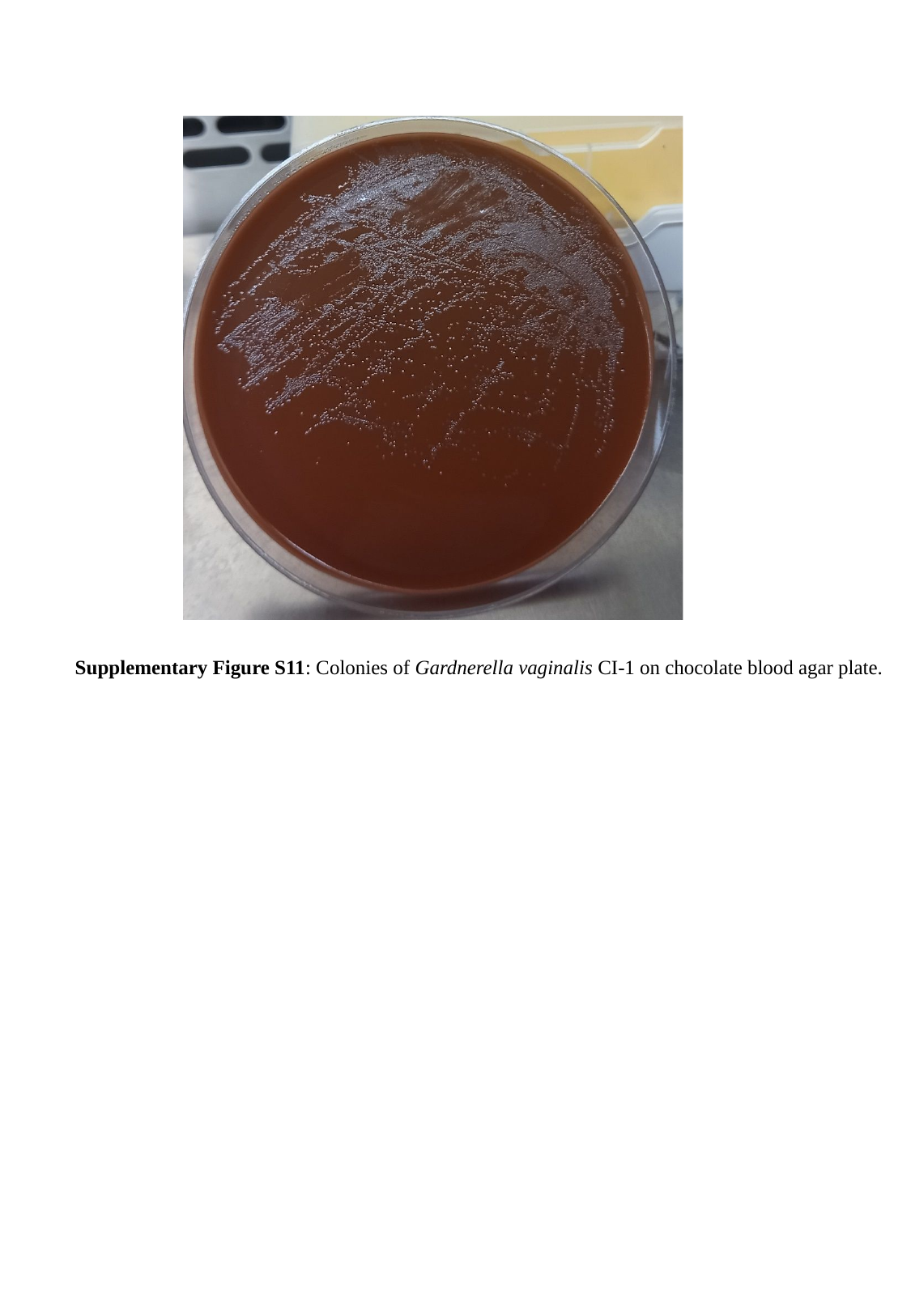

Supplementary Figure S11: Colonies of Gardnerella vaginalis CI-1 on chocolate blood agar plate.

## Slide 15
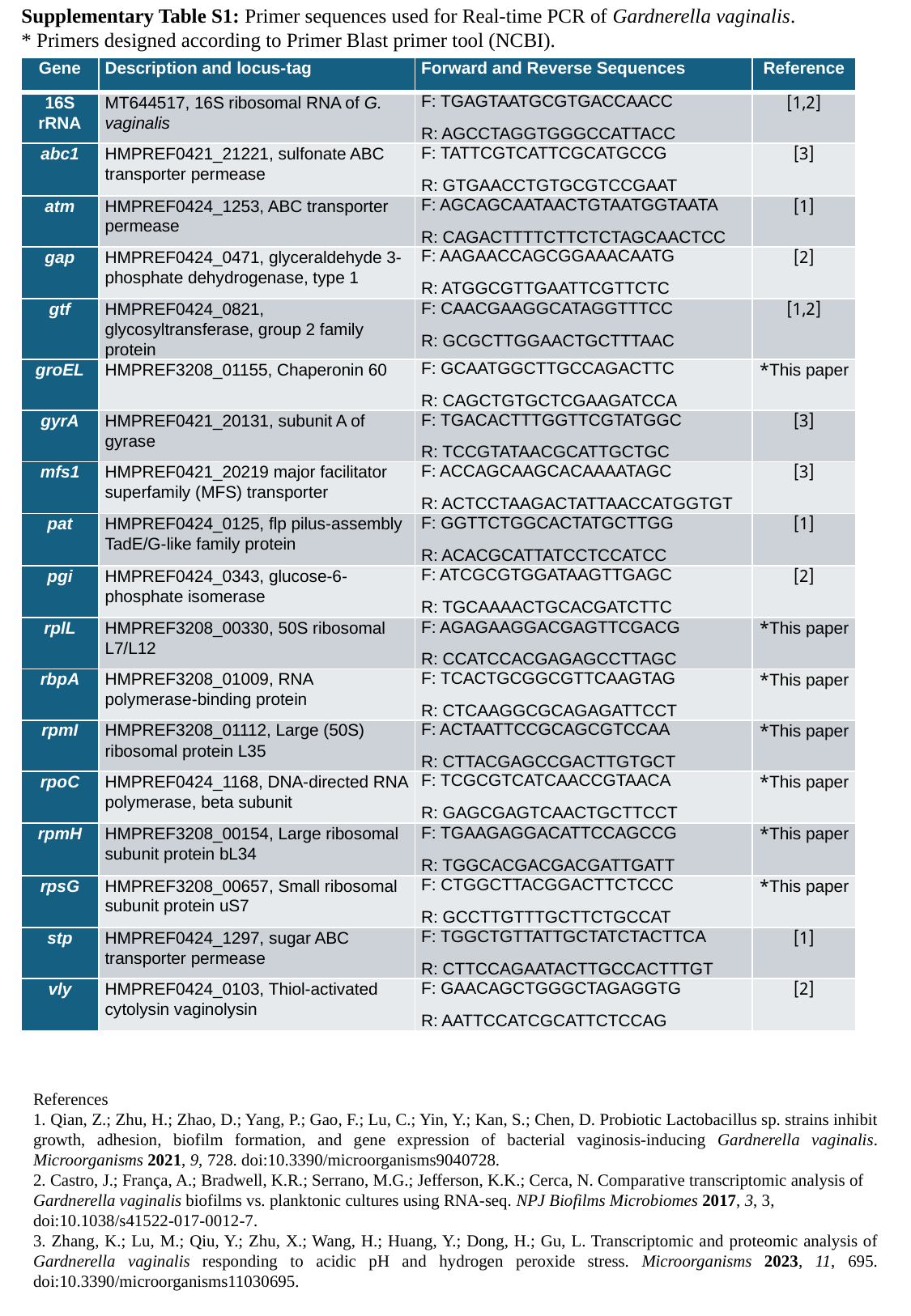

Supplementary Table S1: Primer sequences used for Real-time PCR of Gardnerella vaginalis.
* Primers designed according to Primer Blast primer tool (NCBI).
| Gene | Description and locus-tag | Forward and Reverse Sequences | Reference |
| --- | --- | --- | --- |
| 16S rRNA | MT644517, 16S ribosomal RNA of G. vaginalis | F: TGAGTAATGCGTGACCAACC R: AGCCTAGGTGGGCCATTACC | [1,2] |
| abc1 | HMPREF0421\_21221, sulfonate ABC transporter permease | F: TATTCGTCATTCGCATGCCG R: GTGAACCTGTGCGTCCGAAT | [3] |
| atm | HMPREF0424\_1253, ABC transporter permease | F: AGCAGCAATAACTGTAATGGTAATA R: CAGACTTTTCTTCTCTAGCAACTCC | [1] |
| gap | HMPREF0424\_0471, glyceraldehyde 3-phosphate dehydrogenase, type 1 | F: AAGAACCAGCGGAAACAATG R: ATGGCGTTGAATTCGTTCTC | [2] |
| gtf | HMPREF0424\_0821, glycosyltransferase, group 2 family protein | F: CAACGAAGGCATAGGTTTCC R: GCGCTTGGAACTGCTTTAAC | [1,2] |
| groEL | HMPREF3208\_01155, Chaperonin 60 | F: GCAATGGCTTGCCAGACTTC R: CAGCTGTGCTCGAAGATCCA | This paper\* |
| gyrA | HMPREF0421\_20131, subunit A of gyrase | F: TGACACTTTGGTTCGTATGGC R: TCCGTATAACGCATTGCTGC | [3] |
| mfs1 | HMPREF0421\_20219 major facilitator superfamily (MFS) transporter | F: ACCAGCAAGCACAAAATAGC R: ACTCCTAAGACTATTAACCATGGTGT | [3] |
| pat | HMPREF0424\_0125, flp pilus-assembly TadE/G-like family protein | F: GGTTCTGGCACTATGCTTGG R: ACACGCATTATCCTCCATCC | [1] |
| pgi | HMPREF0424\_0343, glucose-6-phosphate isomerase | F: ATCGCGTGGATAAGTTGAGC R: TGCAAAACTGCACGATCTTC | [2] |
| rplL | HMPREF3208\_00330, 50S ribosomal L7/L12 | F: AGAGAAGGACGAGTTCGACG R: CCATCCACGAGAGCCTTAGC | This paper\* |
| rbpA | HMPREF3208\_01009, RNA polymerase-binding protein | F: TCACTGCGGCGTTCAAGTAG R: CTCAAGGCGCAGAGATTCCT | This paper\* |
| rpmI | HMPREF3208\_01112, Large (50S) ribosomal protein L35 | F: ACTAATTCCGCAGCGTCCAA R: CTTACGAGCCGACTTGTGCT | This paper\* |
| rpoC | HMPREF0424\_1168, DNA-directed RNA polymerase, beta subunit | F: TCGCGTCATCAACCGTAACA R: GAGCGAGTCAACTGCTTCCT | This paper\* |
| rpmH | HMPREF3208\_00154, Large ribosomal subunit protein bL34 | F: TGAAGAGGACATTCCAGCCG R: TGGCACGACGACGATTGATT | This paper\* |
| rpsG | HMPREF3208\_00657, Small ribosomal subunit protein uS7 | F: CTGGCTTACGGACTTCTCCC R: GCCTTGTTTGCTTCTGCCAT | This paper\* |
| stp | HMPREF0424\_1297, sugar ABC transporter permease | F: TGGCTGTTATTGCTATCTACTTCA R: CTTCCAGAATACTTGCCACTTTGT | [1] |
| vly | HMPREF0424\_0103, Thiol-activated cytolysin vaginolysin | F: GAACAGCTGGGCTAGAGGTG R: AATTCCATCGCATTCTCCAG | [2] |
References
1. Qian, Z.; Zhu, H.; Zhao, D.; Yang, P.; Gao, F.; Lu, C.; Yin, Y.; Kan, S.; Chen, D. Probiotic Lactobacillus sp. strains inhibit growth, adhesion, biofilm formation, and gene expression of bacterial vaginosis-inducing Gardnerella vaginalis. Microorganisms 2021, 9, 728. doi:10.3390/microorganisms9040728.
2. Castro, J.; França, A.; Bradwell, K.R.; Serrano, M.G.; Jefferson, K.K.; Cerca, N. Comparative transcriptomic analysis of Gardnerella vaginalis biofilms vs. planktonic cultures using RNA-seq. NPJ Biofilms Microbiomes 2017, 3, 3, doi:10.1038/s41522-017-0012-7.
3. Zhang, K.; Lu, M.; Qiu, Y.; Zhu, X.; Wang, H.; Huang, Y.; Dong, H.; Gu, L. Transcriptomic and proteomic analysis of Gardnerella vaginalis responding to acidic pH and hydrogen peroxide stress. Microorganisms 2023, 11, 695. doi:10.3390/microorganisms11030695.
